# Persistent PirB cleavage drives Golgi-directed trafficking deficits underlying neurodegeneration

**DOI:** 10.1186/s40035-026-00553-5

**Published:** 2026-06-03

**Authors:** Wu-Bo Han, Xian-Dong Liu, Chang-Fei Tang, Junke Zheng, Tian-Le Xu, Nan-Jie Xu, Suya Sun

**Affiliations:** 1 Department of Neurology and Institute of Neurology, Rui Jin Hospital, Shanghai Jiao Tong University School of Medicine, Shanghai, 200025 China; 2 Department of Anatomy and Physiology, Shanghai Jiao Tong University School of Medicine, Shanghai, 200025 China; 3 Songjiang Hospital and Songjiang Research Institute, Shanghai Key Laboratory of Emotions and Affective Disorders, Shanghai Jiao Tong University School of Medicine, Shanghai, 201600 China; 4 Shanghai Key Laboratory of Reproductive Medicine, Shanghai Jiao Tong University School of Medicine, Shanghai, China

## Abstract

**Background:**

Extensive research evidence indicates that neuronal immune receptors play a critical role in Alzheimer's disease (AD). However, it remains unclear how these receptors convert extracellular signals into cellular pathological changes. Proteolytic cleavage of membrane receptors serves as an unconventional pathway for reprogramming cellular functions, and the cleaved fragments often play important roles within the cell. Whether immune receptors mediate their effects in AD through this cleavage-dependent signaling mechanism remains to be clarified.

**Methods:**

We performed proteomic screening of human cerebrospinal fluid to identify cleaved membrane proteins. Cleavage events were validated in postmortem AD brains, primary neurons, and APP/PS1 mice by immunoprecipitation, liquid chromatography-tandem mass spectrometry (LC–MS/MS), and immunofluorescence. Functional impacts were assessed through cathepsin D maturation assays, Retention Using Selective Hooks (RUSH) systems, live imaging of axonal transport, and behavioral tests (e.g., Barnes maze and fear conditioning). Therapeutic potential was evaluated by inhibiting the paired immunoglobulin-like receptor B (PirB) cleavage and overexpression of the GAT domain of Golgi-associated, gamma adaptin ear-containing, ARF binding protein 3 (GGA3).

**Results:**

This study identified a pathogenic proteolytic pathway in AD patients and mouse models, which cleaves PirB, a mouse ortholog of human leukocyte immunoglobulin-like receptor B2 (LILRB2), upon Aβ exposure, generating a C-terminal fragment (PirB-CTF) that accumulated in the Golgi apparatus via retrograde transport. PirB-CTF bound to the GAT domain of GGA3, disrupting Golgi transport, impairing lysosomal maturation, and compromising anterograde synaptic vesicle transport. Inhibiting PirB cleavage or overexpressing GGA3-GAT restored Golgi function, reduced Aβ plaque burden and tau phosphorylation, and rescued memory deficits.

**Conclusion:**

Our study revealed a non-canonical pathway in which proteolytic cleavage repurposes PirB into an intracellular disruptor of Golgi trafficking, directly coupling immune receptor activation to organelle dysfunction in AD. The PirB-CTF/GGA3 interface represents a promising therapeutic target for mitigating trafficking deficits and cognitive decline in neurodegenerative disorders.

**Graphical abstract:**

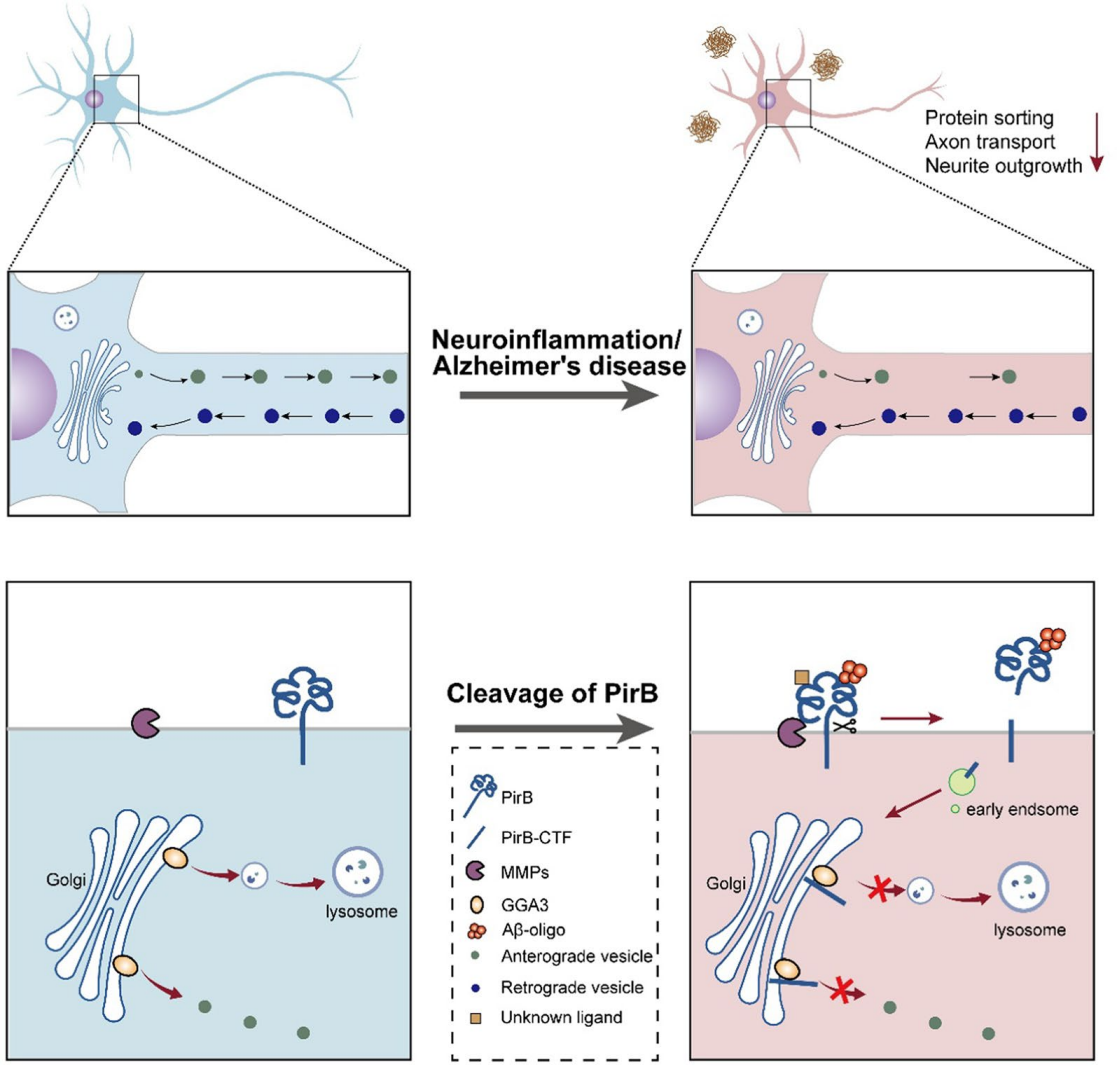

**Supplementary Information:**

The online version contains supplementary material available at 10.1186/s40035-026-00553-5.

## Background

Alzheimer's disease (AD) exhibits profound changes in brain microenvironment, including the occurrence of metabolic stress and chronic neuroinflammation. Membrane receptors play pivotal roles in these processes. While receptors traditionally mediate intercellular communication through ligand-induced conformational changes and downstream signaling cascades, emerging evidence reveals a non-canonical regulatory mechanism, proteolytic cleavage-mediated receptor reprogramming [1–3]. This process generates extracellular and intracellular fragments that dynamically regulate neuronal signaling. Proteolytic cleavage serves as a molecular switch, not only terminating a receptor's canonical function but also endowing the fragments with novel biological activities. The fragments translocate to specific organelles, such as the Notch intracellular domain translocating to the nucleus or the amyloid precursor protein (APP)-derived peptides translocating to mitochondria, where they modulate neuronal survival, synaptic plasticity, and inflammatory responses [4, 5]. This post-translational modification is governed by the activity of proteolytic enzymes, including γ-secretase, α-secretase and matrix metallopeptidases (MMPs) [6–8], as well as ligand binding [9]. Receptors such as TREM2 (triggering receptor expressed on myeloid cells 2) and APP undergo cleavage to release fragments that modulate neuroinflammation or amyloidogenesis [7, 10].

Despite these paradigm-shifting discoveries, systematic profiling of cleavable receptors in neurological contexts remains limited. Notably, several immune inhibitory receptors recently implicated in AD risk are expressed in brain circuits [11–13]. The basic function of these receptors is to maintain cells in a resting state or inhibit cellular biological process to reach cellular homeostasis [14–16]. They regulate innate immune responses and phagocytosis, maintaining an immune homeostasis state and suppressing excessive pro-inflammatory reaction under pathological condition [17, 18]. The inhibitory receptors on neurons participate in the regulation of neural development and synaptic plasticity, as well as in neural activity [19, 20]. However, compared to their well-characterized roles in the immune system, the intracellular signaling and functions of these receptors in the brain are less clear.

In the present study, we identify leukocyte immunoglobulin like receptor B2 (LILRB2, murine homolog, paired immunoglobulin-like receptor B, PirB) as a potential substrate for ligand-induced proteolysis. However, the proteolytic processing of PirB and its functional consequences in AD remain largely unexplored. Therefore, this study aims to investigate whether the relevant pathologies of AD (such as Aβ or inflammation) can induce the cleavage of PirB, to clarify the molecular mechanism by which this cleavage impairs neuronal function, and to evaluate the therapeutic relevance of this pathway.

## Materials and methods

### Animals

All mice were housed in a specific-pathogen-free (SPF) barrier environment at 23 ± 2 °C with humidity from 40% to 70%. All animal procedures were conducted with approval from the Institutional Animal Care and Use Committee (IACUC) at Shanghai Jiao Tong University School of Medicine (No. JUMC2024-211-A). *PirB* knockout (KO) mice were kindly provided by Professor Junke Zheng (Shanghai Jiao Tong University School of Medicine, China) [21, 22]. Genotyping of the colony was performed according to previously described protocols [22, 23]. APP/PS1 mice were provided by Professor Mathias Jucker from Tübingen University. Both APP/PS1 and *PirB* KO mice used in this study were backcrossed to the 129S2/SvPasCrl (129S) strain background. The ages of the mice used in the experiments are detailed in the text. Only male mice were used in this study. It is unknown whether the findings are relevant for female mice.

### Cell line

HEK293T cells were cultured at 37 °C with 5% CO_2_ in Dulbecco’s Modified Eagle’s Medium (DMEM; BasalMedia, Shanghai, China) supplemented with 10% fetal bovine serum (Corning, New York, NY) and 1% penicillin/streptomycin (Gibco, Waltham, MA). The cells were passaged during the logarithmic growth phase.

### Human brain samples

Frozen human frontal cortex tissues were provided by the Brain Bank and Neurodegenerative Disease Research Center at the University of Science and Technology of China (Anhui, China) and the Human Brain Tissue Resource Library, Shanghai Jiao Tong University School of Medicine (Shanghai, China). The non-AD donors included 2 females and 4 males, aged 75, 78, 79, 81, 83 and 85 years, respectively. The AD donors included 3 females, aged 70, 80, and 93 years. There was no significant age difference between the AD and the non-AD groups (*P* = 0.8687). All samples tested negative for HIV and hepatitis. The study was approved by the Ethical Committee of Ruijin Hospital Affiliated to Shanghai Jiao Tong University School of Medicine (Approval No. 2021-214).

### DNA constructs

Mouse *PirB* (accession #: NM_011095.2), *Gga3* (accession #: NM_173048.4), *Vps51* (accession #: NM_001081041.1), *Arcn1* (accession #: NM_145985.4) and *Npy* (neuropeptide Y, accession #: NM_023456.3) genes were amplified from mouse spleen and hippocampal cDNA by PCR. The cDNA of PirB, GGA3, Vps51, ARCN1 were cloned into pLenti-CMV-GFP Puro vector (Addgene, Watertown, MA). The cDNA of Npy was cloned into PCMV-C-mCherry vector (D2628, Beyotime, Shanghai, China). All the mutants were generated from full-length PirB and GGA3. For PirB, four mutants were designed: PirB-mut1 (M630A/S631A); PirB-mut2 (T629A/M630A); PirB-mut3 (P628A/T629A); PirB-mut4 (P627A/P628A). These mutations were introduced by site-directed mutagenesis using the full-length PirB plasmid as the template. Overlapping primers containing the desired mutations were used for PCR amplification, followed by digestion of the methylated template DNA with Dpn I (R0176V, New England Biolabs, Ipswich, MA) at 37 °C for 2 h. The digested product was transformed into *E. coli* DH5α competent cells (CW0808S, CWBIO, Shanghai, China). Positive clones were selected and verified by Sanger sequencing (SAIHENG Biotechnology, Shanghai, China). For GGA3, deletion mutants lacking specific domains (VHS, GAT, GAE, or the hinge region) were generated using a fusion PCR approach. Briefly, overlapping PCR fragments flanking the deletion site were amplified from the GGA3 plasmid template and subsequently fused by a second round of PCR. The resulting fusion product was subcloned into the pLenti-CMV-GFP Puro vector. Furthermore, cDNA fragments encoding the VHS, GAT, GAE, or the hinge domains of human GGA3 and C-terminal fragment (CTF) of PirB (PirB-CTF) were amplified from their respective plasmid templates (GGA3 or PirB). These fragments were also subcloned into the pLenti-CMV-GFP Puro vector. All constructs were confirmed by Sanger sequencing.

### RNAi constructs

The sequences of the shRNAs were as follows: GAC ATG TCT TCC CTG AAT TTA (shHrs-1), ATG ATG AAG TGG CCA ACA AAC (shHrs-2), AGA GGC AAG TGG AAG TTA ATG (shHrs-3); CGT GTG GAC TAC GTC GAT AAA (shVps35-1), CCA AAT CTT GAG TCC AGT GAA (shVps35-2), GCT GTC ACC AAA GAG TTA CTA (shVps35-3); GCC CAA GTC AAT GAA AGT GAA (shGga3-1), ACT GTC AAT ACG GTC ATTA (shGga3-2), ATA AGA GGC GGA CGC TGT TTA (shGga3-3). The target sequences were inserted into the lentivirus by OBiO Technology Company (Shanghai, China).

### Reagents

The following reagents were used: poly-D-lysine (Sigma-Aldrich, Burlington, MA, P0899), lipopolysaccharide (LPS) (Sigma-Aldrich, L2880), Lipofectamine 2000 (Thermo Fisher Scientific, Waltham, MA, 11668019), protein G magnetic beads (Thermo Fisher Scientific, 10004D), anti-HA affinity beads (Chuzhi Biotechnology, Shanghai, China, SA068005), Ni–NTA agarose (Thermo Fisher Scientific, R90101), TAPI-1 (Selleck, Houston, TX, S7434), DAPT (Sigma-Aldrich, D5942), LY2811376 (Clinisciences, Nanterre, France, HY-10472), β amyloid 1–42 (Sigma-Aldrich, AG968), C4d (Abcam, Cambridge, UK, ab198640), GM6001 (Sigma-Aldrich, 364205), biotin (Sigma-Aldrich, 14400), DMSO (Sigma-Aldrich, 472301), B27 (Life Technologies, Carlsbad, CA, 17504044), MMP2 (MedChemExpress, Princeton, NJ, HY-P73810) and MMP9 (MedChemExpress, HY-P70351A).

### Biological process enrichment analysis

The gene annotation enrichment analysis tool DAVID v6.8 was used for Gene Ontology (GO) biological process analysis, based on proteins and protein fragments identified from human cerebrospinal fluid (CSF) samples through mass spectrometry (MS), Olink, and SomaScan. To identify proteins undergoing proteolytic cleavage, we overlapped this subset with proteins located at the cell membrane (i.e., those containing transmembrane domains or glycosylphosphatidylinositol anchors) based on UniProt annotations. For the MS analysis, we selected proteins that reached an enriched level (fold change ≥ 1.5). All protein IDs were converted to Entrez gene IDs to avoid nomenclature discrepancies. We used *H. sapiens* as the background list for screening cleavage proteins and *Mus musculus* for the MS analysis. The negative logarithm (–log10) of the Benjamini-corrected *P*-values was calculated, and the top GO terms for biological processes (GOTERM_BP_DIRECT) were visualized in a graph.

### Neuronal culture

Hippocampal neuron cultures were prepared from postnatal day 0 (P0) *PirB* wild-type (WT) or KO mice, as well as embryonic day 18 (E18) mice (wide type for purifying neurons), as described previously [24]. Mouse brains were removed and transferred into DMEM medium. Hippocampi were dissected out and the meninges were removed. Cleaned hippocampi were minced into small pieces and digested using papain (1 mg/mL; Sigma-Aldrich) and deoxyribonuclease I (5 mg/mL; Sigma-Aldrich) at 37 °C for 30 min. The tissue was then pipetted into a single-cell suspension, followed by centrifugation to remove the digestion solution. The cell pellets were re-suspended in a feeding medium consisting of neurobasal medium supplemented with B27 (Life Technologies), 0.5 mM glutamine, and penicillin/streptomycin. Neurons were then plated onto coverslips coated with poly-*D*-lysine (0.2 mg/mL; Sigma-Aldrich). Half of the feeding medium was replaced every 3 days until the neurons were harvested.

### Neuronal transfection

Primary hippocampal neurons (24-well plates) were transfected on day 3 in vitro (DIV3). 1 μg plasmids (PirB, PirB-mut, PirB-CTF, NPY-mCherry or GGA3-GAT, see DNA constructs section) were mixed with 0.3 M CaCl_2_ (12.5 μL) and 2 × HBS solution (12.5 μL). After replacing the feeding medium with transfection medium (neurobasal medium with 1% penicillin/streptomycin, 500 μL), the plasmid mixture was added, and the neurons were incubated at 37 °C for 60 min. The transfection medium was then replaced with acidified medium pre-treated with CO_2_. After 20 min of incubation, the acidified medium was replaced with feeding medium.

For lentivirus transfection, viruses (OBiO Technology) encoding control (GFP), full-length PirB (PirB-FL), mutant PirB (PirB-mut), and PirB-CTF were transduced overnight in feeding medium. Primary neurons were plated in 24-well plates at a density of 50,000 cells per well. After 72 h, the neurons were treated with lentiviruses at MOIs (multiplicities of infection) of 2 or 3, retaining half the volume of feeding medium. Following overnight incubation with the virus, the feeding medium was removed, and the neurons were washed three times with warm neurobasal medium. The retained medium was then added back for subsequent culture.

### Astrocyte and microglia culture

P0 pups were sacrificed, and their brains were removed and dissected in phosphate-buffered saline (PBS). The dissected cortices were chopped into small pieces and incubated in DMEM containing 0.25% trypsin and 0.05% deoxyribonuclease I at 37 °C for 15 min. The tissue was then pipetted into a single-cell suspension, followed by centrifugation to remove the digestion solution. The cell pellets were re-suspended in DMEM supplemented with 10% fetal bovine serum and plated in T75 culture flasks pretreated with poly-*D*-lysine. The flasks were incubated for 14 days in a humidified incubator at 37 °C with 5% CO_2_. The flasks were shaken mechanically at 220 rpm. The cells remaining attached to the flask were identified as astrocytes, while the suspended cells were microglia. The isolated astrocytes and microglia cells were further cultured for 48 h, and then were lysed for immunoprecipitation.

### Immunofluorescence

For cell immunofluorescence, cells were washed with PBS 3 times and fixed in a solution of 4% formaldehyde and 4% sucrose in PBS for 15 min. Fixed cells were then permeabilized and blocked with 10% donkey serum and 0.1% Triton X-100 in PBS for 30 min at room temperature. Following this, cells were incubated overnight at 4 °C with primary antibodies. After washes with PBST (0.1% Tween-20 in PBS) for 3 × 10 min, the cells were incubated with Alexa Fluor secondary antibodies (1:500) for 60 min at room temperature. The cells were washed in PBST for 3 × 10 min. Finally, the coverslips were mounted onto slides and stored at − 20 °C until confocal imaging.

For brain slice immunofluorescence, mice were anesthetized with 4% isoflurane in oxygen at a flow rate of 1 L/min in an induction chamber. The depth of anesthesia was confirmed by the absence of a withdrawal response to toe pinch. Mice were then transferred to a nose cone for maintenance. The maintenance concentration was adjusted to 2% isoflurane in oxygen at a flow rate of 1 L/min. Mice were perfused with 0.1 M PBS followed by 4% paraformaldehyde (PFA) in phosphate buffer. The brains were removed, post fixed for 48 h, and sectioned at 30 μm. The brain slices were permeabilized and blocked with 10% donkey serum and 0.1% Triton X-100 in PBS for 30 min at room temperature. Slices were incubated overnight at 4 °C with primary antibodies. After washes with PBST (0.1% Tween-20 in PBS) for 3 × 10 min, the brain slices were incubated with Alexa Fluor secondary antibodies (1:500) conjugated to fluorophores for 120 min at room temperature. The brain slices were washed in PBST three times, covered with cover glass and stored at − 20 °C until confocal imaging. Photographs were collected using a Leica SP8 confocal microscope. Images were analyzed by ImageJ (NIH).

The primary antibodies included goat anti-PirB-ecto (1:1000, R&D Systems, Minneapolis, MN, AF2754), mouse anti-Hemagglutinin (HA) (1:1000, Abmart, Shanghai, China, M20003), rabbit anti-Flag (1:1000, Abmart, TT0053), rabbit anti-TGN46 (1:1000, Abcam, ab16059), rabbit anti-HA (1:1000, Cell Signaling Technology, Danvers, MA, 3724), rabbit anti-GM130 (1:1000, Abcam, ab52649), rabbit anti-calnexin (1:1000, Abcam, ab22595), rabbit anti-clathrin (1:1000, Cell Signaling Technology, 4796), rabbit anti-beta COP (1:1000, Abcam, ab2899), rabbit anti-Rab9 (1:1000, Cell Signaling Technology, 5118), rabbit anti-Rab7 (1:1000, Cell Signaling Technology, 9367), rabbit anti-S6 Ribosomal Protein (1:1000, Cell Signaling Technology, 2217), rabbit anti-EEA1 (1:1000, Cell Signaling Technology, 2411), rabbit anti-G3BP (1:1000, Abcam, ab56574), rabbit anti-LAMP1 (1:1000, Abcam, ab24170), goat anti-ionized calcium binding adaptor molecule 1 (Iba1) (1:500, Abcam, ab5076), rabbit anti-glial fibrillary acidic protein (GFAP) (1:1000, Cell Signaling Technology, 12389) and anti-β-amyloid, 1–16 antibody (1:1000, Biolegend, San Diego, CA, SIG-39300).

### Western blotting and immunoprecipitation

Western blotting was performed as previously described [25]. Hippocampal regions from WT, *PirB* KO, LPS-treated, and APP/PS1 mice at various ages or stages were dissected, and homogenized in lysis buffer (50 mM Tris–HCl, pH 7.5; 200 mM NaCl; 5 mM MgCl_2_; 1% NP-40; 10% glycerol; 1 mM DTT; 1 mM PMSF; 50 mM NaF; 1 mM Na_3_VO_4_; and protease inhibitors) at 4 °C for 30 min. Primary hippocampal neurons (on DIV14) and HEK293T cells were also collected, homogenized, and solubilized in the same lysis buffer. The protein lysates were separated by SDS-PAGE, transferred to PVDF membranes, and immunoblotted with primary antibodies. Blots were quantified using Image J. The density of each band was normalized to GAPDH or corresponding loading control signals in the same sample. The primary antibodies included rat anti-ILT4 (Immunoglobulin-like transcript 4, aliases of Lilrb2) (1:500, Santa Cruz Biotechnology, Dallas, TX, sc-53594), goat anti-LILRB3 (1:500, Santa Cruz Biotechnology, sc-9608), mouse anti-tau5 (1:1000, Thermo Fisher Scientific, AHB0042), goat anti-Iba1 (1:1000, Abcam, ab5076), rabbit anti-GFAP (1:1000, Cell Signaling Technology, 12389), mouse anti-HA (1:1000, Abmart, M20003), rabbit anti-cathepsin D (CTSD) (1:1000, Abcam, ab75852), rabbit anti-Flag (1:1000, Abmart, TT0053), rabbit anti-ptau-S199 (1:1000, Abcam, ab4749), rabbit anti-ptau-S404 (1:1000, Abcam, ab92676), mouse anti-ptau-AT8 (1:200, Thermo Fisher Scientific, MN1020), mouse anti-ptau-S396 (1:1000, Thermo Fisher Scientific, 44-752G), goat anti-PirB-ecto (1:1000, R&D Systems, AF2754), anti-β-Amyloid, 1–16 (1:1000, Biolegend, SIG-39300), rabbit anti-Golgin-97 (1:1000, Cell Signaling Technology, D8P2K), rabbit anti-Hrs (1:1000, Cell Signaling Technology, D7T5N), rabbit anti-vps35 (1:1000, Cell Signaling Technology, E6S4I), rabbit anti-GGA3 (1:1000, Cell Signaling Technology, 4367) and mouse anti-GAPDH (1:3000, Invitrogen, MA515738).

For immunoprecipitation, human brain sample, mouse hippocampal tissue, primary cultured neurons, astrocytes, microglia, or HEK293T cells were homogenized in lysis buffer at 4 °C for 1 h. The samples were then immunoprecipitated with the primary antibodies (1:100) for 4 h and incubated with protein G beads for an additional 3 h at 4 °C. Enriched proteins were separated by SDS-PAGE, transferred to PVDF membranes, and immunoblotted with the primary antibodies. The primary antibodies included mouse anti-ILT4 (1:100, Santa Cruz Biotechnology, Dallas, TX, sc-390287), goat anti-LILRB3 (1:100, Santa Cruz Biotechnology, sc-9608), and goat anti-PirB-ecto (1:100, R&D Systems, AF2754).

### Culture medium concentration

Primary hippocampal neurons were plated in a 10-cm dish at a density of 50,000 cells/cm^2^. The culture medium was collected at DIV14 and centrifuged for 3 min at 12,000 rpm to eliminate suspended cells and cell debris. The supernatant was concentrated using a centrifugal filter (Millipore, Burlington, MA, UFC9003) to a volume of 1 ml. Anti-PirB-ecto antibody (R&D Systems, AF2754, 1:100) was added to enrich PirB-NTF. The enriched PirB-NTF was then detected by the same antibody using Western blotting.

### Isolation of the Golgi apparatus

The Golgi and Golgi-related vesicles were isolated with the Minute Golgi Apparatus Enrichment Kit (GO-037, Invent Biotechnologies, Beijing, China). Unilateral hippocampus from 9-month-old WT mice or APP/PS1 mice were placed at a filter cartridge and grinded in 550 μL Buffer A. The lysate was centrifuged at 16,000 × *g* for 30 s, then for 5 min at 5000 × *g* at 4 °C. The supernatant was transferred to a fresh 1.5-mL tube and centrifuged at 16,000 × *g* for 30 min at 4 °C. Following centrifugation, 400 μL supernatant was transferred to a fresh 1.5-mL tube and 400 μL Buffer B was added. The mixture was placed at 0 °C for 10 min and centrifuged at 8000 × *g* for 5 min at 4 °C. The supernatant containing secretory vesicles and membrane of the trans-Golgi apparatus was transferred to a fresh 1.5 mL tube, and 100 μL buffer D was added. After incubation on ice for 20 min, the supernatant was centrifuge at 16,000 × *g* for 5 min. The pellets containing vesicles and membrane of the trans-Golgi apparatus were resuspended in Denaturing Protein Solubilization Reagent and analyzed by Western blotting. Anti-Golgin-97 and anti-GAPDH were used to verify the efficiency of Golgi apparatus isolation. Anti-Lilrb3 was used to detect the level of PirB-CTF.

### Immunoprecipitation-mass spectrometry analysis

For proteolytic cleavage site search, HEK293T cells were transfected with empty vector (pLenti-CMV-GFP Puro) or vector carrying HA-tagged PirB (PirB-HA). Forty-eight hours after transfection, cells were collected, homogenized at 4 °C for 1 h in lysis buffer, and immunoprecipitated with anti-HA beads for 4 h at 4 °C. After several washes with lysis buffer, the immunoprecipitates were eluted from the beads and subjected to SDS-PAGE. The gels were stained using Silver Staining Kits (Beyotime, P0017S), and PirB-CTF band was carefully excised and digested with trypsin. The resulting tryptic peptides were subsequently analyzed by liquid chromatography-tandem mass spectrometry (LC–MS/MS).

To screen for novel binding partners, HEK293T cells were transfected with empty vector (pLenti-CMV-GFP Puro) or vector carrying HA-tagged PirB-CTF (PirB-CTF-HA). Forty-eight hours after transfection, the cells were collected and homogenized in lysis buffer at 4 °C for 1 h. Immunoprecipitation was performed using anti-HA beads for 4 h at 4 °C. After washing with lysis buffer, the beads were mixed with hippocampal homogenates from 8-week-old WT mice and incubated overnight at 4 °C. Following multiple washes with lysis buffer, the immunoprecipitants were eluted from the beads and subjected to SDS-PAGE. The gel was cut for in-gel digestion, and the resulting tryptic peptides were analyzed by LC–MS/MS. The binding partners were ranked in the order of fold change (fold change ≥ 2) in peptide counts of the detected proteins. The raw data for PirB-CTF LC–MS/MS in the hippocampus have been submitted to ProteomeXchange and are available with an identifier PXD063306 (Access details: Log in to the PRIDE website using the following details: Project accession: PXD063306; Token: Ngtu8wBa82RS).

### Aβ oligomer preparation for cell treatment

Aβ oligomers were prepared based on a modified version of a previously established method [13, 26]. Aβ1-42 powder was dissolved in 1, 1, 1, 3, 3, 3-hexafluoro-2-propanol (HFIP) to a final concentration of 1 mM and incubated at room temperature for monomerization. The HFIP was then evaporated under vacuum conditions for 3 h. The remaining Aβ1-42 film was dissolved in freshly prepared dimethyl sulfoxide (DMSO) at 5 mM as a stock solution and stored at − 20 °C. This stock was thawed and diluted in PBS to achieve a final concentration of 100 μM. After sonication for 30 s, the Aβ1-42 solution was incubated at 22 °C for 16 h, followed by incubation at 4 °C for 24 h and centrifugation at 16,000 × *g* for 15 min. The resulting supernatant contained Aβ-oligomers, which were confirmed by Western blot analysis. Aβ-oligomers were added to the culture medium to a final concentration of 100 nM.

### Venus fluorescence complementation assay

For Venus fluorescence complementation assay in HEK293T cell, cells were transfected with PirB-VN and STX6-VC plasmids (custom-synthesized by SAIHENG Biotechnology, Shanghai, China). Seventy-two hours after transfection, DMSO or Aβ oligomers (100 nM) were added. Fluorescence images were then captured at indicated time points. For Venus fluorescence complementation assay in neurons, *PirB*-KO hippocampus neurons were transfected with PirB-VN, STX6-VC and cytoplasmic mCherry plasmids (D2628, Beyotime) at DIV7. Seventy-two hours after transfection, DMSO, Aβ oligomers (100 nM), GM-6001 (1 µM) and 6E10 antibody (BioLegend, SIG-39300, 1:500) were added. Fluorescence images were then captured at indicated time points, and analyzed by Image J. The fluorescence intensities at all time points were normalized to the initial fluorescence intensity.

### LPS treatment

Eight-week-old 129S mice were anesthetized with isoflurane and placed on a stereotaxic apparatus. LPS was dissolved in PBS to a final concentration of 2.5 µg/µL, and 2 µL of LPS was slowly injected into the lateral cerebral ventricle at a rate of 0.1 µL/min. Control group mice received the same volume of PBS. Mice were monitored until they recovered on a thermal blanket set to 34 °C.

Seven days post-injection, the mice were anesthetized and perfused with PBS, followed by 4% PFA in PBS. The brains were immersed in 4% PFA for 48 h, and then cut into 30-µm coronal sections. The sections were stained with rabbit anti-GFAP (1:1000, Cell Signaling Technology, 12389) and goat anti-Iba1 (1:1000, Abcam, ab5076). For immunoprecipitation, hippocampi were dissociated at the indicated time (1, 3, 5, 7 and 14 days after injection).

### Preparation of soluble PirB ectodomain (sPirB)

To generate sPirB, the PirB ectodomain was cloned into the PCMV-C-His vector (D2650, Beyotime) and transfected into HEK293T cells. Both the cells and the media were collected and purified using Ni–NTA agarose (Thermo Fisher Scientific, R90101), following the standard protein purification protocol. The purity of sPirB was assessed by Coomassie brilliant blue staining. sPirB was then used to treat primary neuron cultures at a final concentration of 1 mg/mL.

### Neutralizing peptide preparation

The anti-PirB antibody was incubated with the neutralizing peptide of PirB (from amino acid 777 to 827 of PirB) (90 μM) for 8 h at 4 °C. Then the blocked antibody was incubated with a PVDF membrane (Millipore, ISEQ00010) overnight at 4 °C. After enhanced chemiluminescence detection, the PVDF membrane was incubated with non-blocked anti-PirB antibody overnight at 4 °C.

### MMPs administration

After being transfected with PirB-HA or PirB-mut-HA, HEK293T cells were treated with 1 μg/ml MMP2 or MMP9 for 24 h. Then the cells were harvested and lysed, and subjected to Western blotting.

### RUSH assay with live cell imaging

According the previous research [27], we tagged streptavidin with KEDL (Lys-Asp-Glu-Leu) sequence to serve as an endoplasmic reticulum (ER) hook and fused the target peptide with tdTomato and streptavidin-binding peptide (SBP) as a reporter. These plasmids were custom-synthesized by SAIHENG Biotechnology Company, using pcDNA™3.1( +) vector (Invitrogen, Carlsbad, CA, V790-20). HEK293T cells were transfected with RUSH plasmids alongside plasmids expressing PirB-FL, PirB-mut, PirB-CTF, GAT, or PirB-CTF/GAT (all PirB-related plasmids were equipped with cytoplasmic GFP). The cells were cultured without biotin. Upon addition of biotin (40 μM), live imaging was conducted over 120 min using Olympus FV3000, with scans performed every 5 min.

### Live cell imaging for axon transport

Hippocampal neurons were plated on glass-bottom dishes at a density of 50,000 cells/cm^2^ and imaged using an Olympus FV3000 microscope. The microscope was equipped with a chamber that supplied humidified 5% CO_2_ and maintained a temperature of 37 °C. To monitor axon transport, neurons were transfected with NPY-mCherry (the cDNA of Npy was cloned into the PCMV-C-mCherry vector) and PirB related plasmid (equipped with cytoplasmic GFP) at DIV3. At 48 h post-transfection, cells were imaged and kymographs were generated every 1 s. For neurons with Aβ oligomer treatment, kymographs were captured 4 h after Aβ treatment.

### Duolink proximity ligation assay (PLA)

The Duolink PLA kit was purchased from Sigma-Aldrich. Fixed primary neurons were blocked in blocking solution at 37 °C for 1 h, and incubated with primary antibody at 4 °C overnight. The primary antibodies included rabbit anti-PirB (1:500, Abcam, ab271086), mouse anti-GGA3 (1:500, MABN1556, Sigma-Aldrich) and mouse anti-TGN38 (1:500, MAB7944, R&D Systems). PLA Probe anti-rabbit PLUS (Sigma-Aldrich, DU092002) and PLA Probe anti-mouse MINUS (Sigma-Aldrich, DU092004) were applied and incubated at 37 °C for 1 h. Next, ligation and amplification were conducted using Detection Reagents Red (Sigma-Aldrich, DU092008). Duolink Mounting Medium containing DAPI was used to label the nucleus. Finally, images were captured by a Leica SP8 confocal microscope.

### GST pull-down assay

PirB-CTF-His and GST-fused GGA3, GGA3-ΔGAT, GGA3-GAT fragments were expressed in *E*. *coli* BL21 cells and purified by Ni–NTA agarose (for purifying PirB-CTF-His) and glutathione sepharose (G4501, Millipore) (for purifying GST-fused proteins). The purified PirB-CTF-His was incubated with GGA3, GGA3-ΔGAT, GGA3-GAT or GST proteins and then pulled down with glutathione sepharose. The bound proteins were eluted from the beads, denatured at 65 °C for 15 min and then subjected to Western blotting.

### Stereotactic injection and intracerebral administration

AAV-EGFP-GAT-Flag virus (serotype 2, titer 1.04 × 10^12^ genomic copies per mL) and AAV-EGFP virus (serotype 2, titer 2.42 × 10^12^ genomic copies per mL) were packaged by OBiO Technology Co. Ltd. (Shanghai, China). Six-month-old WT and APP/PS1 mice received stereotactic injection and intracerebral administration. The mice were anesthetized and mounted on a stereotactic apparatus. The coordinates for bilateral hippocampal injection (AP/ML/DV from Bregma) were + 1.8/ ± 1.5/ − 1.9 mm, in a volume of 1.5 µL. The position and effects of viral injection were assessed three weeks later.

### Barnes maze test (BMT)

BMT was conducted on a circular platform that is 100 cm in diameter and has 20 identical holes (4 cm in diameter), of which only one was an escaping hole. On training days (days 1–4), mice were allowed to explore on the platform for 3 min to find the escaping hole. They were guided to the escaping hole if they did not find the escaping hole after 3 min. On test day (day 5), the time to find the escaping hole was recorded. Spatial memory was assessed as the latency to find the hiding box (days 1–4) and the latency to reach the edge of escaping hole (day 5).

### Fear conditioning test

Fear conditioning test was conducted using the Ugo Basile Fear Conditioning System (Ugo Basile, Varese, Italy). The conditioning chamber (17 cm × 17 cm × 25 cm) was equipped with stainless steel shocking grids connected to a precision feedback current-regulated shocker. On the day before conditioning training, mice (after completion of BMT) were allowed to explore freely for 20 min in the chamber with wallpapers (context A). On the training day, each mouse was placed in context A and allowed to explore it freely for 2 min. Then a pure tone (Conditioned Stimulus, CS) was played for 18 s, followed by an electric foot shock (US, 0.75 mA, 2 s duration) through the floor grid. The conditioned mouse was returned to the home cage 30 s after completion of electric shock. The floor and walls of the cage were cleaned with 75% ethanol before next trial. Twenty-four hours after conditioning, the mouse was placed in context A, and the freezing time was recorded to assess contextual fear memory. To test auditory fear memory, the mouse was placed in a test chamber with a non-shocking floor and a different wallpaper (context B), and allowed to explore it freely for 2 min. Then the tone was played continually for 1 min. The freezing time during tone presentation in context B was recorded to assess auditory fear memory.

### Statistical analysis

Statistical analyses were performed using GraphPad Prism (GraphPad Software Inc.). Data are presented as mean ± SEM. Unpaired *t*-test was used for comparison between two independent groups. One-way ANOVA followed by a post-hoc test was used for multiple groups with single variance. For comparisons involving two groups with multiple variables, two-way ANOVA followed by a post-hoc test was conducted. Statistical methods, number of *n* replicates and *P* values are annotated in figure legends. Statistical significance was defined as *P* < 0.05. All statistical tests were two-sided.

## Results

### Ligand-dependent proteolysis of PirB by MMPs in AD

**Fig. 1:**
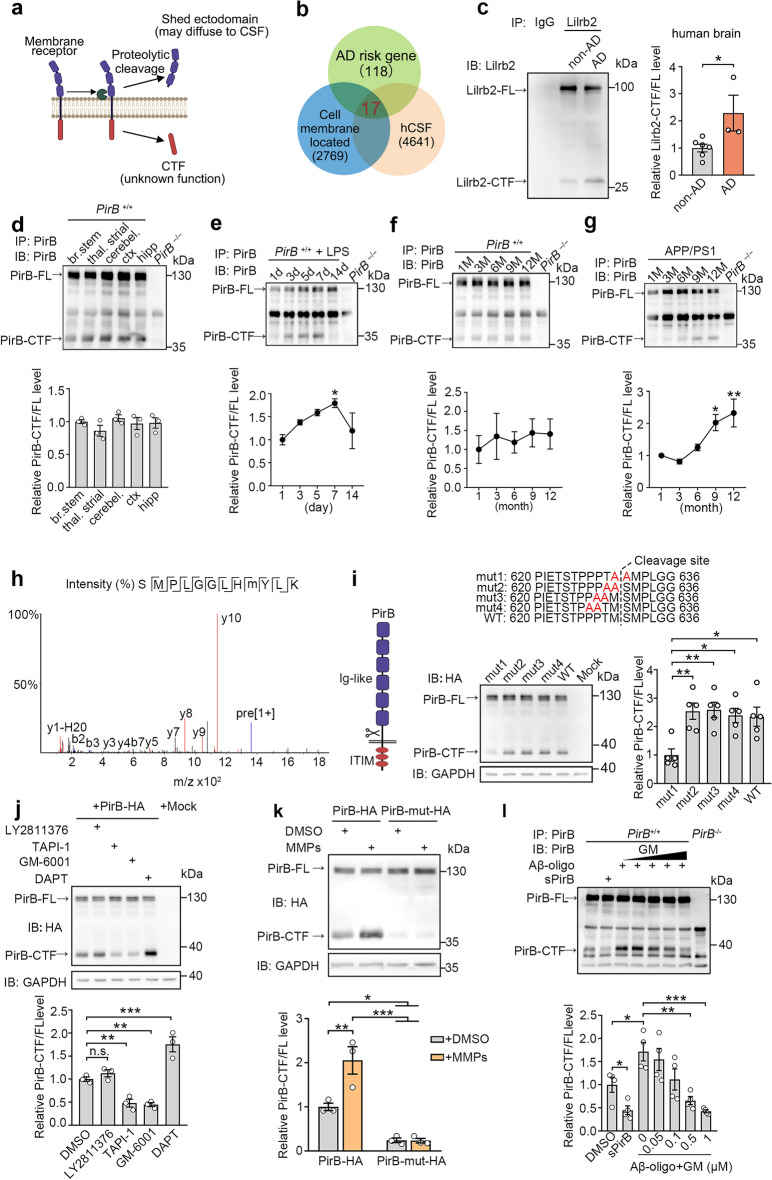
Ligand-dependent proteolysis of PirB by MMPs in physiological and pathological processes. **a** Schematic of the strategy for screening out the proteins that undergo proteolytic cleavage in the central nervous system. **b** Venn diagram showing that 17 AD risk gene products may undergo proteolytic cleavage. **c** Full-length (LILRB2-FL) and C-terminal fragment of LILRB2 (LILRB2-CTF) in the frontal cortex tissues from AD patients are enriched by mouse anti-LILRB2, followed by rat anti-LILRB2 Western blot. The level of LILRB2-CTF was normalized to LILRB2-FL (*n* = 3 AD and 6 non-AD, unpaired *t*-test, *P < 0.05). **d** PirB-FL and PirB-CTF immunoprecipitate from brain stem (br. stem), thalamus and striatum (thal. striat), cerebellum (cerebel.), cortex (ctx) and hippocampus (hipp) of 8-week-old mice are detected. *PirB* knockout (KO) mice were used as control (*n* = 3 mice per group, one-way ANOVA). **e** Levels of PirB-FL and PirB-CTF in mouse hippocampus at 1, 3, 5, 7 and 14 days after LPS administration (*n* = 3 mice per group, one-way ANOVA with Dunn’s post-hoc test, *P < 0.05). **f** Levels of PirB-FL and PirB-CTF in WT mouse hippocampus by immunoprecipitation at 1, 3, 6, 9 and 12 months of age (*n* = 4 mice per group, one-way ANOVA). **g** Levels of PirB-FL and PirB-CTF in the hippocampus of APP/PS1 mice by immunoprecipitation at 1, 3, 6, 9 and 12 months of age (*n* = 4 mice per group, one-way ANOVA with Dunn’s post-hoc test, *P < 0.05, **P < 0.01). All PirB-CTF levels were normalized to PirB-FL. **h** Liquid chromatography-tandem mass spectrometry identified the extracellular juxta-membrane region peptide sequence of PirB-CTF. **i** Western blot identifying the proteolytic cleavage site of PirB (*n* = 5 biological replicates in each group, one-way ANOVA with Dunn’s post-hoc test, *P < 0.05, **P < 0.01). **j** PirB cleavage was prevented by incubation with TAPI-1 (an inhibitor of several proteases) and GM-6001 (a general MMP inhibitor), but not LY2811376 (a β-secretase inhibitor), and enhanced by DAPT (a γ-secretase inhibitor) (*n* = 3 biological replicates in each group, one-way ANOVA with Dunn’s post-hoc test, **P < 0.01, ***P < 0.001). **k** MMPs (1 μg/mL MMP2 + 1 μg/mL MMP9) treatment enhanced the cleavage of PirB but not PirB-mut (*n* = 3 biological replicates in each group, two-way ANOVA with Tukey’s post-hoc test, *P < 0.05, **P < 0.01, ***P < 0.001). **l** Endogenous PirB cleavage was prevented by blocking of ligand binding (*n* = 4 biological replicates in each group, DMSO versus sPirB, unpaired* t*-test, *P < 0.05); and GM-6001 decreased PirB cleavage upon Aβ oligomer stimulation (*n* = 4 biological replicates in each group, one-way ANOVA with Dunn’s post-hoc test, *P < 0.05, **P < 0.01, ***P < 0.001)

To identify membrane proteins undergoing proteolytic processing in the human brain, we analyzed human CSF datasets [28] containing secreted proteins and cleavage fragments originating from membrane proteins. We utilized UniProt annotations to isolate the membrane proteins from this dataset and identified 842 membrane proteins with cleavage signatures (Fig. S1a, b). These proteins were enriched in pathways linked to synaptic and axonal regulation, such as cell adhesion and axon guidance (Fig. S1c). Analysis of gene–disease associations for the 842 proteins revealed an enrichment of several neurological disorders, including epilepsy and AD (Fig. S1d). To examine the potential association between protein cleavage and AD, we cross-referenced the list of 842 proteins with known AD risk genes [29–33]. Among AD risk genes, 17 of 118 (~ 14.4%) encode cleaved membrane proteins detected in human CSF, including LILRB2/PirB, an immune receptor implicated in Aβ oligomer binding (Fig. 1a, b and Fig. S1e).

Previous studies have found that LILRB2/PirB is expressed in brain and functions by binding to ligands such as major histocompatibility complex class I (MHC-I) and Aβ oligomers [13, 34]. However, it is unclear whether it undergoes protein cleavage and whether the cleaved fragments exert cellular functions. To investigate this, we first detected whether LILRB2 can be cleavage to produce a CTF in brain samples of AD patients. Given the low expression level in the central nervous system, we performed immunoprecipitation to enhance detection. Strikingly, the level of LILRB2-CTFs relative to full-length LILRB2 (LILRB2-FL) was significantly elevated in AD brains compared with the controls (Fig. 1c). Then we validated the presence of PirB-CTF in mouse brain using an antibody targeting the intracellular domain. In mice, PirB-CTF (~ 40 kDa) was detected in multiple brain regions as well as in primary neurons and astrocytes (Fig. 1d and Fig. S1f, g). We also detected PirB N-terminal fragment (PirB-NTF) in extracellular supernatant (Fig. S1h), indicating that the cleavage process occurs primarily at the cell membrane.

We next investigated whether neuroinflammation is involved in the cleavage of PirB. In adult WT mice, following LPS-induced acute neuroinflammation (Fig. S1i), PirB cleavage peaked at day 7 post-treatment (Fig. 1e). Furthermore, the PirB-CTF levels showed progressive accumulation with age in APP/PS1 mice. However, this pattern was not observed in adult WT mice, which showed constant PirB-CTF levels across age (Fig. 1f, g). These results indicate that PirB cleavage is dynamically regulated in a context-dependent manner and suggest its potential relevance to AD progression.

To determine the cleavage site of PirB, PirB-HA (HA at C terminus) was overexpressed in HEK293T cells, and was enriched using an HA-tag antibody (Fig. S2a). Silver staining revealed efficient enrichment of both PirB-FL and PirB-CTF (Fig. S2b). Subsequently, the sequence of PirB-CTF was analyzed by LC–MS/MS. The MS data indicated that PirB might be cleaved between methionine (M630) and serine (S631) within the extracellular juxtamembrane region (Fig. 1h and Fig. S2c). To validate this, mutations were introduced around the putative cleavage site. Compared to other mutations, substitution of M630 and S631 with Alanine (A) resulted in a significant decrease in the level of PirB-CTF (Fig. 1i). To identify the protease responsible for PirB cleavage, we treated cells with specific protease inhibitors. We found that PirB cleavage was significantly inhibited by a broad-spectrum protease inhibitor TAPI-1 and an MMP inhibitor GM-6001, but unaffected by the β-secretase inhibitor LY2811376. Interestingly, treatment with DAPT, a γ-secretase inhibitor, significantly increased the level of PirB-CTF, suggesting that PirB-CTF is a substrate of γ-secretase (Fig. 1j). To further confirm that MMPs can mediate PirB cleavage, we added a mixture of MMP-2 and MMP-9. The administration of MMPs significantly enhanced the cleavage of PirB, and this effect was absent in the cleavage-resistant PirB mutant (PirB-mut) (Fig. 1k).

To verify whether the cleavage of PirB is dependent on ligands like Notch1, we isolated sPirB [35] that can antagonize the ligand of PirB (Fig. S2e, f). Adding sPirB to primary cultured neurons effectively mitigated PirB cleavage, confirming the ligand-dependent cleavage of PirB (Fig. 1l). We then generated Aβ1-42 oligomers to investigate the impact of ligand stimulation on PirB-CTF production (Fig. S2d). Treatment with Aβ oligomers led to a significant elevation in PirB-CTF level, and this effect was attenuated by GM-6001 or TAPI-1 in a dose-dependent manner (Fig. 1l and Fig. S2g). We also found that another ligand of PirB, C4d, which binds at the same site as Aβ oligomers [36], could promote PirB cleavage (Fig. S2h, i). These results demonstrate that PirB undergoes MMP-dependent cleavage at the extracellular juxtamembrane region, a process potentiated by ligand binding.

### PirB-CTF is transported retrogradely to the Golgi apparatus via endosomes

To gain insights into the function of PirB-CTF, we next investigated its cellular localization, as the distribution of proteins governs their functions. We overexpressed PirB-CTF-HA in primary cultured hippocampal neurons from *PirB* KO mice and performed immunostaining for HA and various organelle markers. Single panel confocal imaging analysis revealed PirB-CTF enrichment in the trans-Golgi networks (Fig. S3a–c).

**Fig. 2:**
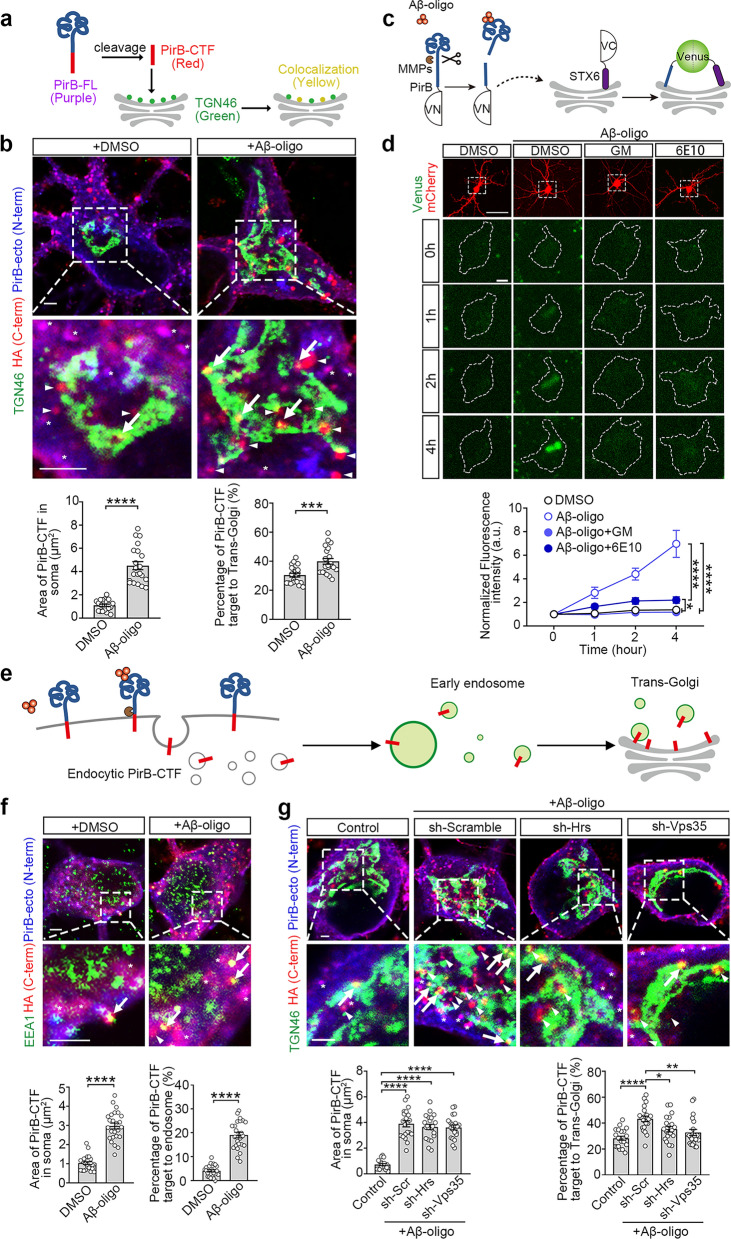
PirB-CTF is retrogradely transported to the Golgi apparatus via endosomes. **a** Schematic of immunofluorescence staining strategy. **b** Single-plane confocal imaging shows the subcellular location of PirB-CTF in primary *PirB* KO mouse hippocampal neurons. HA (red, arrowheads) and PirB N-terminus (blue) for PirB-FL (purple, asterisks); HA only for PirB-CTF (red); and TGN46 (green) for trans-Golgi. The yellow puncta (arrows) indicate PirB-CTF located at trans-Golgi. The panels below show the quantitative analysis of the areas of PirB-CTF puncta in the soma and the ratios of PirB-CTF area occupied in trans-Golgi (*n* = 21 neurons from 3 biological replicates per group, unpaired *t*-test, ***P < 0.001, ****P < 0.0001). Scale bars: 2 μm. **c** Schematic of fluorescence complement experiment. The N-terminal fragment of the Venus (VN) is fused to PirB and the C-terminal fragment of the Venus (VC) is fused to Golgi protein STX6. When PirB-VN undergoes cleavage, the PirB-CTF-VN targets to the Golgi apparatus, where the venus fragments reconstruct to generate fluorescence. **d** Representative complementation fluorescence images of PirB-VN and STX6-VC and images of cytosolic mCherry in hippocampal neurons. The time course of complementation fluorescence was quantified (*n* = 7–9 neurons from 3 biological replicates per group, two-way ANOVA with Turkey’s post-hoc test, *P < 0.05, ****P < 0.0001). 6E10, Aβ antibody. GM, GM-6001. Scale bars: 50 μm (upon), 5 μm (below). **e** Schematic of PirB-CTF retrograde transport to trans-Golgi. **f** Single-plane confocal imaging shows the subcellular location of PirB-CTF in early endosomes in *PirB* KO primary hippocampal neurons. HA (red, arrowhead) and PirB N-terminus (blue) for PirB-FL (purple, asterisk), HA only for PirB-CTF (red), and EEA1 (green) for endosomes. The yellow puncta (arrows) indicate PirB-CTF localized at early endosomes. Scale bars: 5 μm. The area of PirB-CTF puncta in soma and the ratio of PirB-CTF area occupied in endosomes were quantified (*n* = 25 neurons from 3 biological replicates per group, unpaired *t*-test, ****P < 0.0001). **g** Single-plane confocal imaging shows the subcellular location of PirB-CTF in *PirB* KO primary hippocampal neurons with Aβ oligomer treatment. HA (red, arrowhead) and PirB N-terminus (blue) for PirB-FL (purple, asterisk), HA only for PirB-CTF (red), and TGN46 (green) for trans-Golgi. The yellow puncta (arrows) indicate PirB-CTF located at trans-Golgi. Scale bars: 5 μm. The area of PirB-CTF puncta in soma and the ratio of PirB-CTF area occupied in trans-Golgi were quantified (*n* = 21 neurons from 3 biological replicates per group, one-way ANOVA with Tukey’s post-hoc test, *P < 0.05, **P < 0.01, ****P < 0.0001)

To further confirm this finding under physiological conditions, we transfected PirB-FL-HA into the *PirB* KO neurons and performed immunostaining for the HA tag, PirB ectodomains (PirB-ecto) and the trans-Golgi apparatus marker TGN46. The co-localization of the HA tag and the PirB-ecto immunofluorescence signal indicates PirB-FL (purple), while the HA-tag signal (red) alone represents PirB-CTF (Fig. 2a). We found that the level of PirB-CTF was increased significantly and more co-localization of PirB-CTF and TGN46 was observed after Aβ oligomer stimulation (Fig. 2b).

To confirm these findings, a fluorescence complementation assay was performed [37]. The N-terminal fragment of the Venus fluorescent protein was fused to the C-terminus of PirB (PirB-VN), and its complementary C-terminus was fused to the C-terminus of syntaxin6 (STX6-VC), a marker of the trans-Golgi apparatus. When the two fusion proteins come into close proximity, the two halves of the Venus fluorophore would recombine and generate fluorescence (Fig. 2c). We first verified this method on HEK293T cells. Stimulation with Aβ oligomers significantly enhanced the signal of Venus (Fig. S3d, e). We then co-transfected these two plasmids along with mCherry into the *PirB* KO neurons, and observed that induction with Aβ oligomers resulted in a notable increase of fluorescence intensity within the transfected neurons. This effect was blocked by the presence of both the Aβ antibody 6E10 and the MMP inhibitor GM-6001, implying production of PirB-CTF and its translocation to the Golgi apparatus under Aβ oligomer stimulation (Fig. 2d). Meanwhile, we also detected increased endogenous PirB-CTF on the isolated Golgi apparatus from AD mice (Fig. S3f). These data indicate that the cleavage product PirB-CTF targets into the Golgi apparatus in vitro and in vivo.

Although the transmembrane domain is preserved in PirB-CTF, which would typically localize it on the plasma membrane, we observed its accumulation in the Golgi apparatus. The way of PirB-CTF transportation to the Golgi apparatus is still unclear. It is known that membrane proteins can be endocytosed into endosomes and transported retrogradely from endosomes to trans-Golgi network through the endosomal sorting complexes required for transport (ESCRT) pathway and the retromer complex [38–41] (Fig. 2e). We first assessed colocalization of PirB-CTF with endosomal marker EEA1 (Fig. 2f), and then used shRNAs to knockdown some key components of these pathways such as Hrs for the ESCRT pathway [42] and Vps35 for retromer complex [41, 43] (Fig. S4a, b). We found that knockdown of these components could reduce the accumulation of PirB-CTF in the Golgi apparatus without affecting the cleavage of PirB (Fig. 2g). These findings suggest that PirB-CTF is retrogradely transported to the Golgi apparatus via endosomal pathways dependent on ESCRT and retromer complex.

### PirB-CTF disrupts the Golgi-directed trafficking

**Fig. 3:**
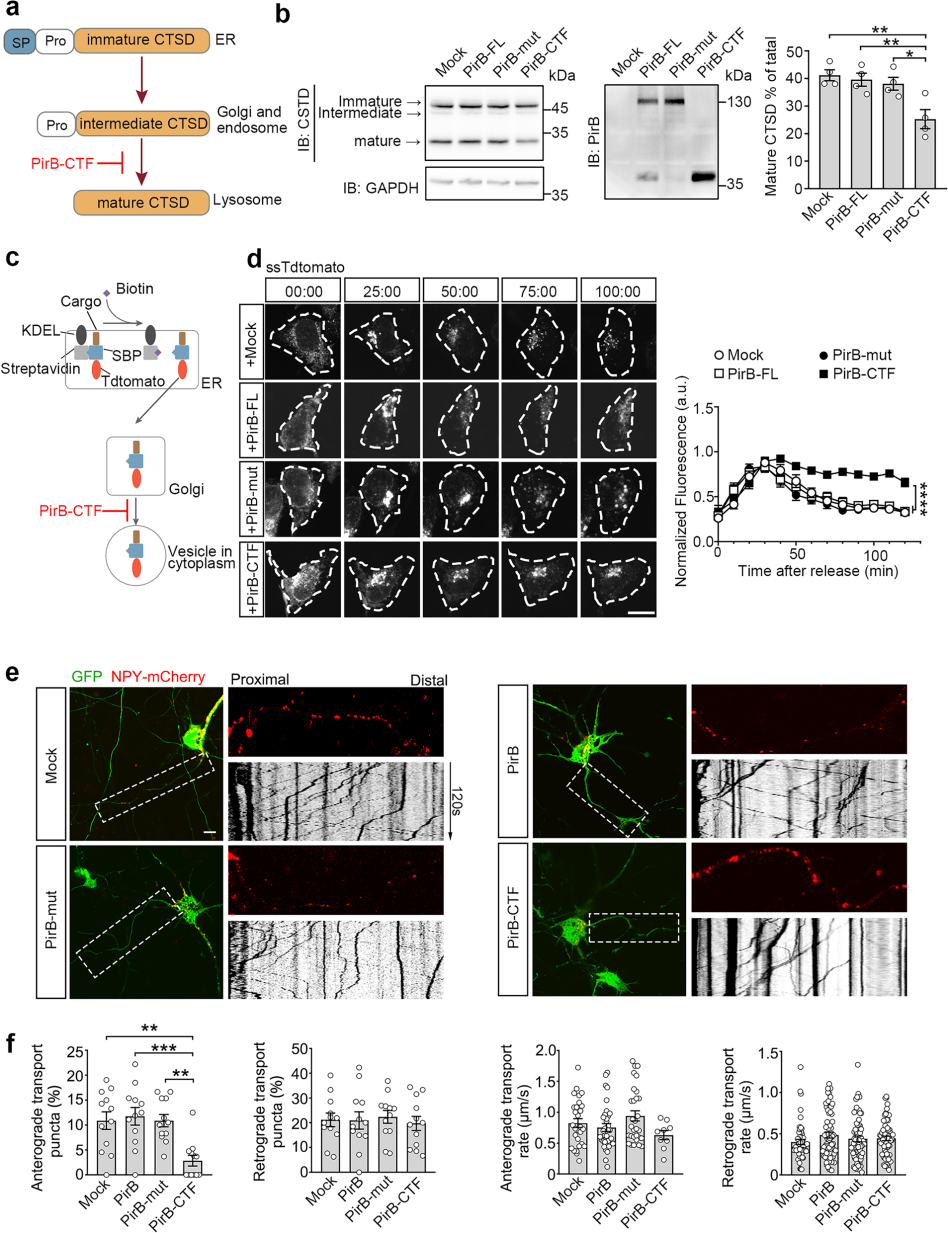
PirB-CTF impairs protein transport function of Golgi apparatus. **a** Schematic of CTSD trafficking and maturation. **b** Measurement of maturation of CTSD in primary cultured *PirB* KO hippocampal neurons transfected with empty vector, PirB-FL, PirB-mut and PirB-CTF (no HA tag) for 72 h (*n* = 4 biological replicates per group, one-way ANOVA with Sidak’s post-hoc test, *P < 0.05, **P < 0.01). **c** Schematic of the RUSH system principle. **d** Trafficking of RUSH-ssTdtomato co-expressed with empty vector, PirB-FL, PirB-mut and PirB-CTF in HEK293T cells upon biotin addition. The time of biotin addition was defined as 00:00. Scale bar: 10 μm. The plot shows normalized fluorescence intensity in the Golgi apparatus (*n* = 10 cells from 3 biological replicates per group, two-way ANOVA with Turkey’s post-hoc test, ****P < 0.0001). **e** Monitoring of protein transport function of Golgi apparatus in PirB-FL, PirB-mut and PirB-CTF neurons by NPY-mCherry imaging of axon segment adjacent to the soma. Scale bar: 10 μm. **f** Statistic plots of the percentages and the rates of anterograde and retrograde transport NPY-mCherry puncta (*n* = 12 neurons from 3 biological replicates per group, one-way ANOVA with Turkey’s post-hoc test, **P < 0.01, ***P < 0.001)

Due to the accumulation of PirB-CTF in the Golgi apparatus, we hypothesize that PirB-CTF may regulate the functions of the Golgi apparatus. First, we examined the maturity of CTSD as an indicator for protein sorting function of Golgi apparatus. The maturation of CTSD undergoes three distinct processes: an immature form within the ER, an intermediate form within the Golgi apparatus, and a mature form within the lysosome (Fig. 3a). Impaired Golgi function will inhibit the entry of the intermediate CTSD into the lysosomes, resulting in decreased proportion of the mature form of CTSD [44]. Our results showed that overexpression of PirB-CTF in primary cultured *PirB* KO hippocampal neurons, but not PirB-FL or the cleavage-insensitive PirB-mut, led to a reduction of mature CTSD (Fig. 3b and Fig. S4c). Considering that Aβ itself can damage the function of the Golgi apparatus [45], we used C4d, another ligand of PirB, to increase the level of endogenous PirB-CTF in primary cultured WT hippocampal neurons [36]. We verified that administration of C4d could promote the production of PirB-CTF, which inhibited the maturation of CTSD (Fig. S4d). The results indicated that accumulation of PirB-CTF specifically influences the protein sorting function of the Golgi apparatus.

To validate the secretory traffic of the Golgi apparatus, we employed the RUSH system [27], which contains two fusion proteins, a streptavidin-fused KEDL hook and an SBP-fused ssTdtomato reporter. Upon addition of biotin, a nontoxic vitamin that competitively binds streptavidin, the hook and the reporter complexes retained in ER are dissociated, thereby allowing the reporter to be released from the ER and resume its transport to the final compartment (Fig. 3c). We transfected HEK293T cells with RUSH-related plasmids and PirB-FL, PirB-mut, or PirB-CTF respectively. After addition of biotin, a slower diffusion rate of fluorescence in the Golgi apparatus was observed only in PirB-CTF cells (Fig. 3d, and Movie S1). This observation suggests that PirB-CTF impairs the secretory function of the Golgi apparatus.

As the primary site and center of vesicle generation and trafficking, the Golgi apparatus plays a critical role in axon transport and neurite outgrowth [46–49]. We therefore hypothesized that PirB-CTF may affect axonal transport. We measured the trafficking of NPY-mCherry, a marker for secretory vesicles [50], in the axon region adjacent to the neuronal soma in *PirB* KO hippocampal neurons. We found that the proportion of anterograde transport NPY puncta was decreased in the presence of PirB-CTF, whereas the retrograde transport proportion and the rate of puncta remained unchanged (Fig. 3e, f, and Movie S2). The attenuated axonal transport may influence neurite outgrowth. Neurons expressing PirB-CTF showed slower neurite growth during development stage. However, PirB-FL or PirB-mut also impaired the neurite outgrowth (Fig. S4e, f). This may be due to the ability of PirB *per se* to promote actin depolymerization [13, 51]. These results indicate that accumulation of PirB-CTF in the Golgi apparatus causes damage to its function.

### Interactions between PirB-CTF and GGA3 mediate Golgi dysfunction

**Fig. 4:**
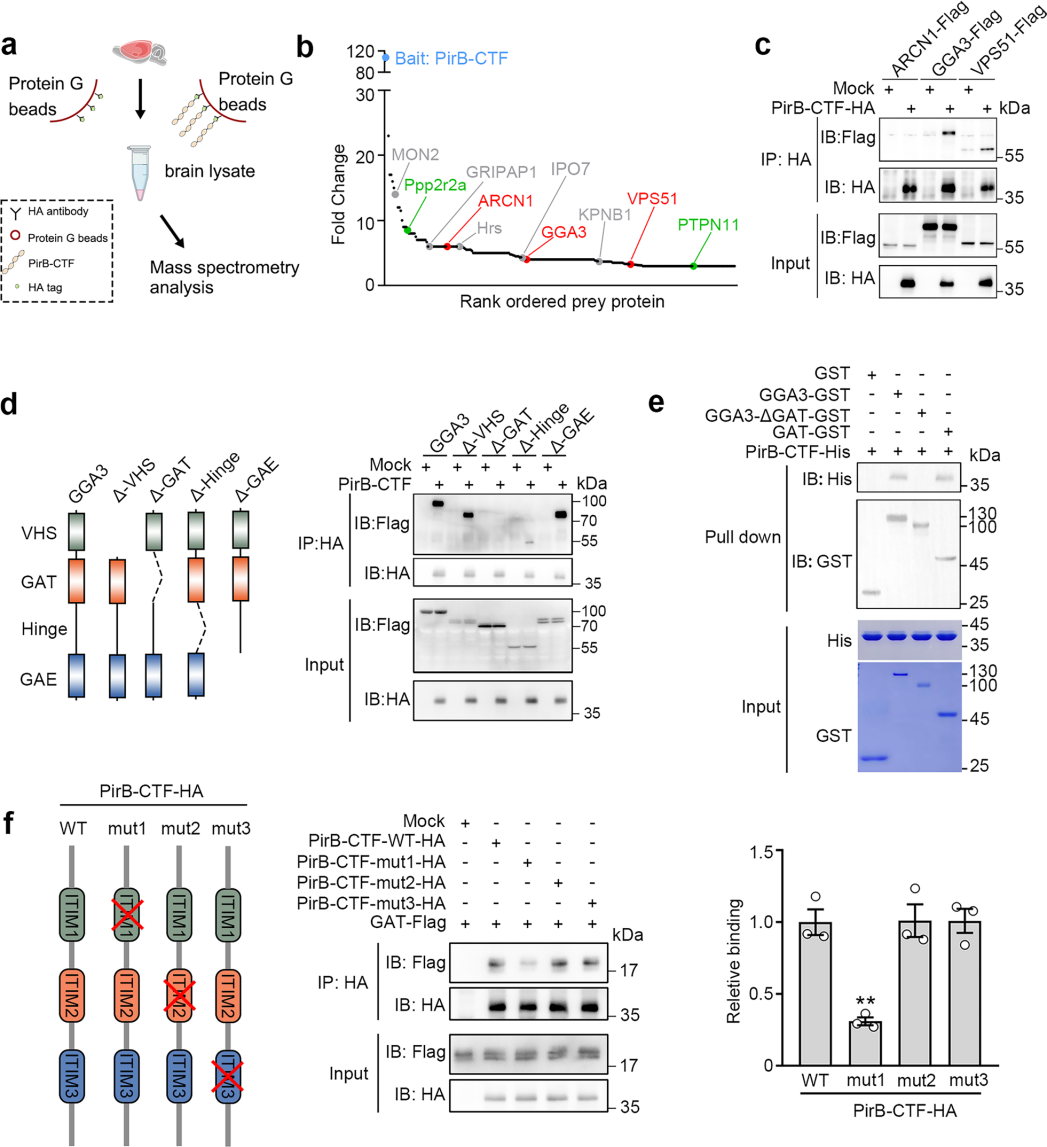
GGA3 is a novel binding partner of PirB-CTF. **a** Flowchart of experimental strategy for searching novel binding partners of PirB-CTF. **b** Prey proteins detected by LC–MS/MS (666 proteins) are ranked in the order of spectral count fold change. Proteins with a function in protein transport are labeled in gray, PirB-binding proteins Ppp2r2a and PTPN11 are highlighted in green, and proteins located in Golgi apparatus are highlighted in red. **c** Co-immunoprecipitation (Co-IP) of exogenously expressed PirB-CTF-HA with ARCN1-Flag, GGA3-Flag or VPS51-Flag in HEK293T cells. **d** Co-IP of exogenously expressed PirB-CTF-HA with Flag-tagged domain-lacking mutants of GGA3 in HEK293T cells. **e** GST pull-down assay showing interactions between PirB-CTF and GAT domain. Coomassie brilliant blue staining shows the purity of the GST-fusion proteins and the His-tag protein. **f** Co-IP of exogenously expressed GAT-Flag and HA-tagged ITIM-motif-lacking mutants of PirB-CTF in HEK293T cells

To identify the Golgi apparatus-associated proteins that interact with PirB-CTF, we enriched HA-tagged PirB-CTF from HEK293T cells and then incubated PirB-CTF with mouse hippocampal lysate. The binding partners were analyzed by LC–MS/MS (Fig. 4a). GO analysis of enriched binding partners indicated that PirB-CTF may play a role in regulating protein transport, which is consistent with our previous findings (Fig. S5a, b). Proteomic analysis identified Golgi-associated proteins (ARCN1, GGA3, VPS51) as PirB-CTF interactors and the enriched positive controls PTPN11 and PP2A subunit ppp2r2a, corroborating our method [13, 52] (Fig. 4b). To further clarify the binding partners for PirB-CTF, we co-expressed HA-tagged PirB-CTF with Flag-tagged ARCN1, GGA3, or VPS51. We then immunoprecipitated PirB-CTF and detected both GGA3 and VPS51, suggesting that these proteins are binding partners for PirB-CTF (Fig. 4c). The binding of PirB-CTF to VPS51 further confirmed its retrograde transport to the Golgi apparatus via endosomal pathways. Immunofluorescence analysis revealed a co-localization of PirB-CTF with GGA3 but not ARCN1, confirming that GGA3 is a binding partner of PirB-CTF (Fig. S5c).

To further verify the interactions between GGA3 and PirB-CTF, PLA was performed to detect their endogenous interactions in the Golgi apparatus. Under the stimulation of Aβ oligomers, the signal of PLA was enhanced, suggesting this endogenous interaction was increased (Fig. S5d). Furthermore, after downregulating GGA3, the interaction between PirB-CTF and trans-Golgi network showed no change, suggesting that GGA3 may mediate the function of PirB-CTF rather than being involved in regulating the localization of PirB-CTF (Fig. S5e, f). To further explore the binding between PirB-CTF and GGA3 and the function, we expressed various domains (VHS, GAT, hinge and GAE) of GGA3 as well as the domain-lacking mutants in HEK293T cells. Our results indicated that GGA3 bound to PirB-CTF through its GAT domain rather than other domains (Fig. 4d and Fig. S5g). To confirm that PirB-CTF and GAT domain interact directly, we generated GST-tagged GGA3, GGA3-ΔGAT and GAT fragments as well as PirB-CTF-His. We found that PirB-CTF bound directly to GGA3 via the GAT domain (Fig. 4e). The intracellular segment of PirB contains several ITIM motifs, which are necessary for its inhibitory function. Therefore, we hypothesized that PirB-CTF might interact with GGA3-GAT through these ITIM motifs. We overexpressed GGA3-GAT and PirB-CTF mutant lacking ITIM motifs. The result indicated that the first ITIM motif was critical for this interaction (Fig. 4f). These findings demonstrate that GGA3 is a novel binding partner of PirB-CTF, with the GAT domain identified as the binding region.

**Fig. 5:**
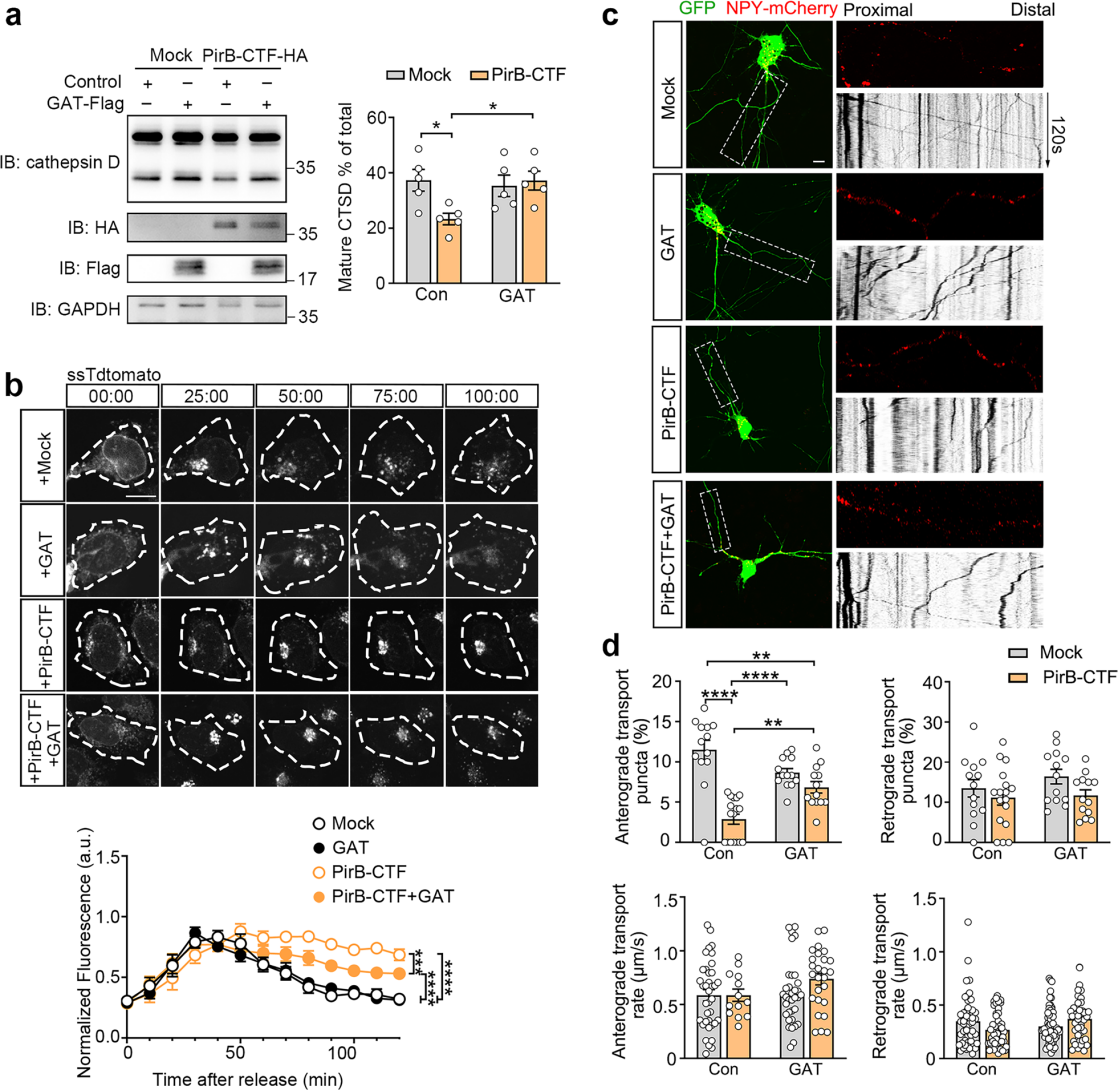
GGA3-GAT can counteract the inhibitory effect of PirB-CTF. **a** Effects of GGA3-GAT overexpression on the maturation of CTSD in *PirB* KO neurons overexpressing PirB-CTF (*n* = 5 biological replicates per group, two-way ANOVA with Turkey’s post-hoc test, *P < 0.05). **b** Imaging showing the effects of GGA3-GAT domain on the trafficking of RUSH-ssTdtomato in the presence of PirB-CTF. The time of biotin addition was defined as 00:00. Scale bar: 10 μm. Plot shows normalized fluorescence intensity in the Golgi apparatus (*n* = 10 cells from 3 biological replicates per group, two-way ANOVA with Turkey’s post-hoc test, ***P < 0.001, ****P < 0.0001). **c** NPY-mCherry imaging of axon segment adjacent to the soma of PirB-CTF overexpressing neurons. The effect of GAT domain on protein transport function of Golgi apparatus was monitored. Scale bar: 10 μm. **d** Quantification of the percentage of anterograde and retrograde transport NPY-mCherry puncta as well as the transport rate (*n* = 13–17 neurons from 3 biological replicates per group, two-way ANOVA with Turkey’s post-hoc test, **P < 0.01, ****P < 0.0001)

To investigate whether modulating the interaction between GGA3-GAT and PirB-CTF could alleviate the inhibitory effect of PirB-CTF, we co-expressed the GAT domain to neutralize PirB-CTF accumulated in the Golgi apparatus. Overexpression of PirB-CTF in *PirB* KO neurons reduced the mature CTSD levels. However, GAT domain overexpression abolished this effect (Fig. 5a). Furthermore, overexpression of GAT alone did not significantly affect the function of the Golgi apparatus, suggesting the specificity of this intervention. Additionally, GAT attenuated the PirB-CTF-induced secretory trafficking deficits (Fig. 5b, and Movie S3). The decreased proportion of anterograde NPY-mCherry puncta caused by PirB-CTF returned to normal level in the presence of GAT domain, whereas the rate and the proportion of retrograde puncta were not influenced by GAT domain (Fig. 5c, d and Movie S4). Consistent with its effects on axonal trafficking, the inhibition of axonal extension mediated by PirB-CTF was also rescued by GAT domain (Fig. S6a, b). These results indicate that the GAT domain alleviated the inhibitory effect of PirB-CTF on Golgi functions.

### Therapeutic targeting of PirB cleavage alleviates AD phenotypes

**Fig. 6:**
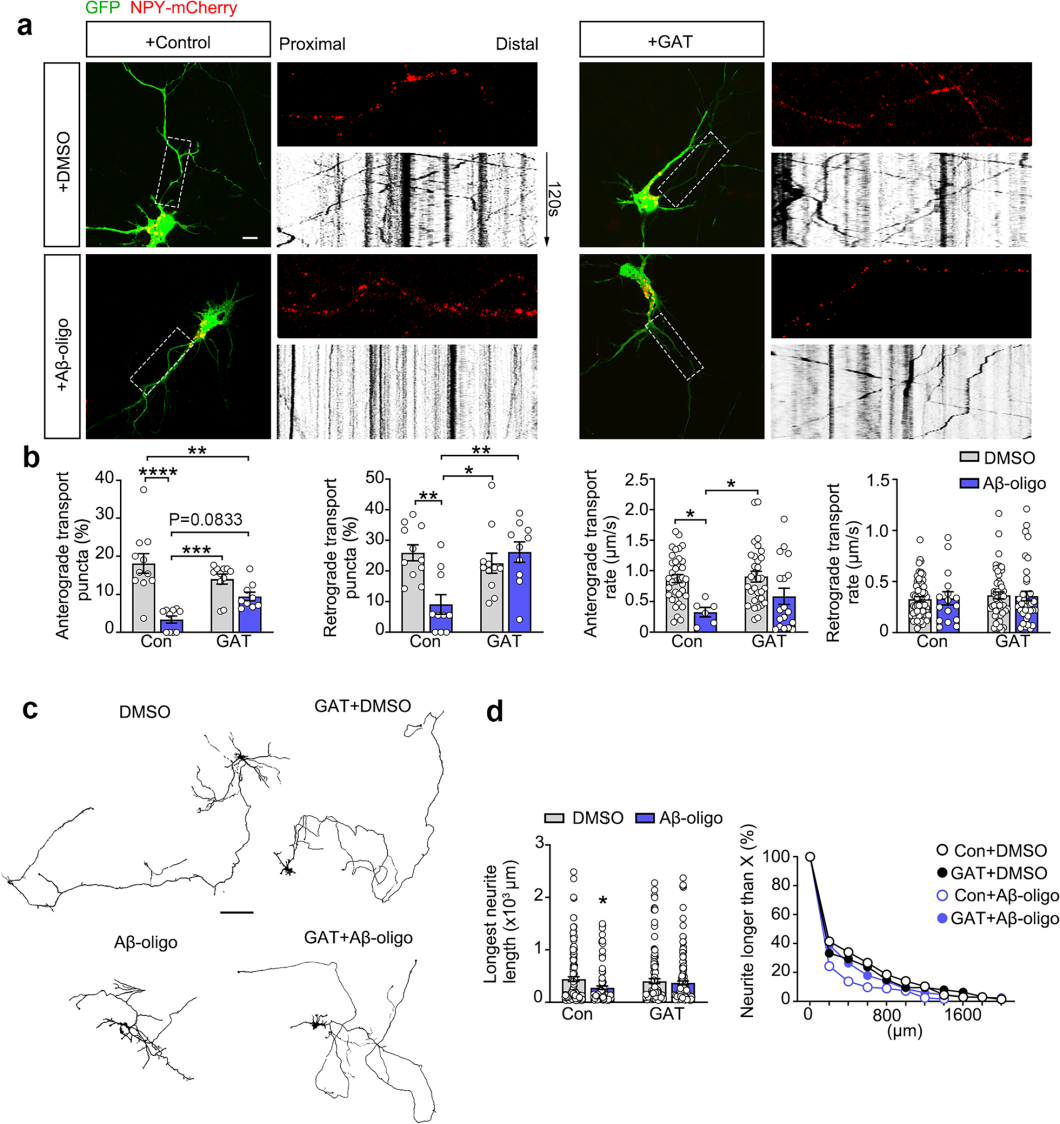
GGA3-GAT alleviates the damage to axonal transport and neural protrusion growth induced by Aβ oligomers. **a** NPY-mCherry imaging of axon segment adjacent to the soma in primary cultured WT mouse hippocampal neurons treated with Aβ oligomers. The effect of GAT domain on protein transport function of Golgi apparatus was monitored. Scale bar: 10 μm. **b** Quantification of the percentage of anterograde and retrograde transport NPY-mCherry puncta as well as the transport rate (*n* = 9–11 neurons from 3 biological replicates per group, two-way ANOVA with Turkey’s post-hoc test, *P < 0.05, **P < 0.01, ***P < 0.001, ****P < 0.0001). **c** Morphological images of primary cultured WT hippocampal neurons treated with Aβ oligomers in the presence or absence of GAT domain. Scale bar: 100 μm. **d** Quantitative analyses of the length of longest neurites of neurons in **c** and the cumulative curves (*n* = 122–151 cells from 5 biological repeats per group, two-way ANOVA with Turkey’s post-hoc test, *P < 0.05)

To further elucidate the functional roles of GAT domain under pathological conditions, we assessed its capacity to neutralize the toxic effects of Aβ oligomers. Addition of Aβ oligomers strongly inhibited anterograde and retrograde axon transport (Fig. 6a, b and Movie S5). This is due to the cytotoxic effects of Aβ oligomers, including microtubule depolymerization and impairment of Golgi apparatus function [45, 53]. Aβ oligomers also significantly inhibited neurite outgrowth (Fig. 6c, d). Overexpression of GAT domain partially reversed these effects, indicating the neutralizing effect of GAT domain on PirB-CTF triggered by Aβ. These findings indicate GAT domain is a critical modulator of PirB-mediated signaling in AD pathological environment, highlighting its potential to preserve neuronal function.

**Fig. 7:**
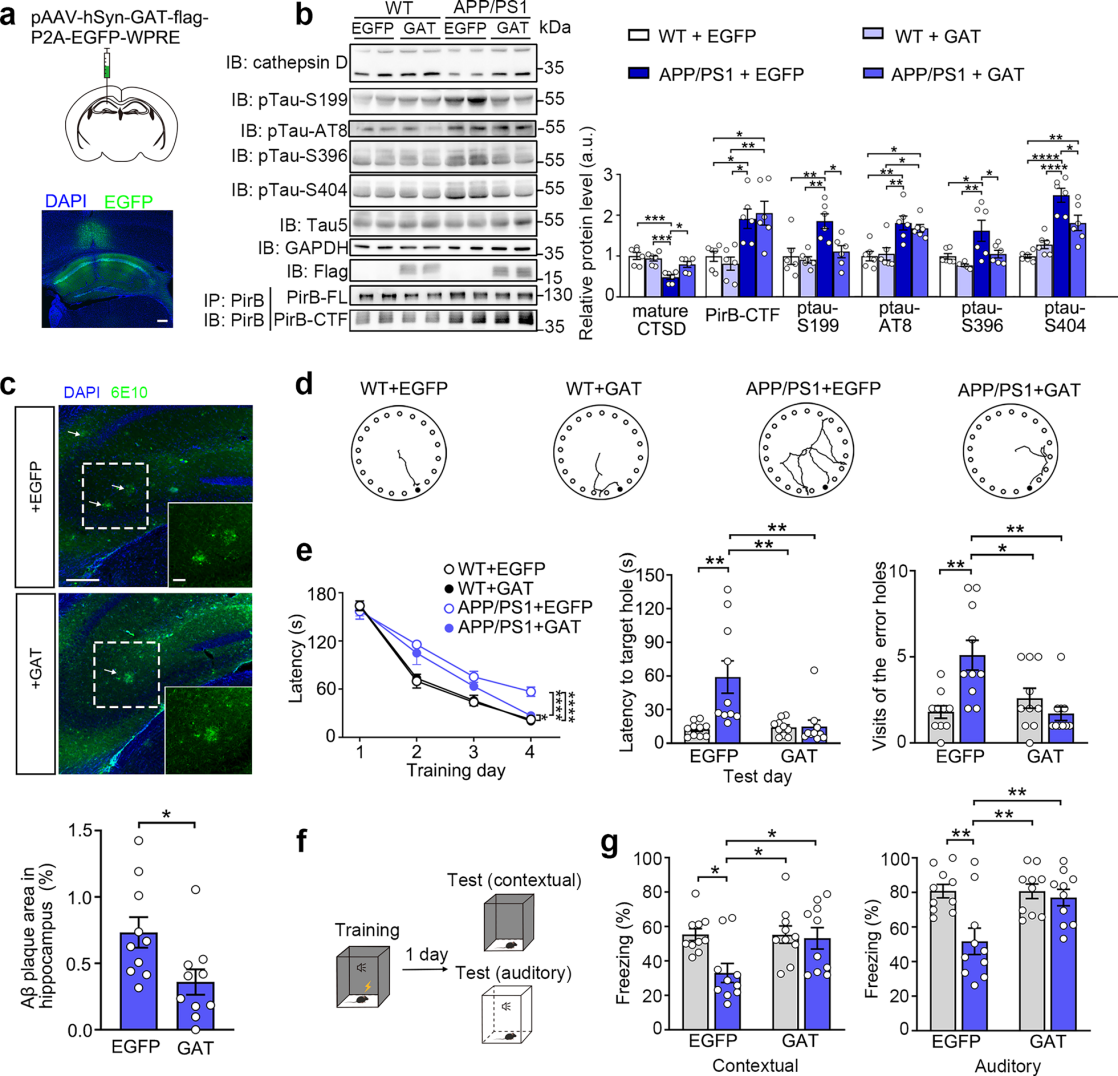
Inhibition of PirB-CTF reverses Golgi dysfunction and behavior deficits in AD mice. **a** Experimental scheme of virus injection in 6-month-old WT or APP/PS1 mouse hippocampus (upper) and histological verification of the site of injection in the hippocampus (lower). Scale bar: 200 μm. **b** Western blotting analysis of mature CTSD, ptau and PirB-CTF in WT and APP/PS1 mouse hippocampus in the presence or absence of the GAT domain (*n* = 6 mice per group, two-way ANOVA with Sidak’s post-hoc test, *P < 0.05, **P < 0.01, ***P < 0.001, ****P < 0.0001). **c** Immunofluorescence images of 6E10 staining in the brain sections of 6-month-old APP/PS1 mice injected with empty EGFP or GAT virus. White arrows denote Aβ plaques. Scale bars: 200 μm (overview) and 50 μm (magnified images). Aβ plaque areas were quantified (*n* = 10 mice per group, unpaired *t*-test, *P < 0.05). **d** Representative BMT tracks on day 5 (test day). **e** The latency to the target hole on days 1–4 (training days) and day 5 (test day) (*n* = 10 mice per group, two-way ANOVA with Turkey’s post-hoc test, *P < 0.05, **P < 0.01, ****P < 0.0001). Visits of error holes were also recorded (*n* = 10 mice per group, two-way ANOVA with Turkey’s post-hoc test, *P < 0.05, **P < 0.01). **f** Experimental scheme of fear conditioning test. **g** Quantification of percentages of freezing time in contextual fear memory test and auditory fear memory test (*n* = 10 mice per group, two-way ANOVA with Turkey’s post-hoc test, *P < 0.05, **P < 0.01)

Since the dysfunction of the Golgi apparatus has been involved in neurodegenerative diseases [45, 54], we investigated whether reversing the Golgi dysfunction caused by elevated PirB-CTF levels could improve the performance of APP/PS1 mice. To explore whether reducing the PirB-CTF level could restore Golgi function in vivo, PirB-CTF generation was suppressed by perfusing GM-6001 into the hippocampus of AD mice to inhibit the activity of MMPs. We found that decreasing the PirB-CTF level effectively restored Golgi function (Fig. S6c, d). Considering that MMP inhibitors may induce broad non-specific effects, we overexpressed PirB or PirB-mut in APP/PS1 mice in the *PirB* KO background to more specifically verify that PirB is a substrate for MMPs (Fig. S6e). We found that PirB-mut did not produce high level of PirB-CTF in the AD pathological condition, restored the impaired Golgi function (Fig. S6f), and exhibited significantly reduced levels of phosphorylated tau and accumulation of Aβ plaque compared to PirB group in AD mice (Fig. S6f, g). We further investigated whether neutralizing the inhibitory effect of PirB-CTF could rescue the performance of AD mice by overexpressing the GAT domain in the hippocampus. Following hippocampal overexpression of the GAT domain, the reduced levels of mature CTSD in APP/PS1 mice were restored (Fig. 7a, b). Meanwhile, phosphorylated tau levels and Aβ plaque burden were significantly reduced (Fig. 7b, c). We also found that the GAT domain overexpression shortened the latency to find the target hole in the BMT and improved performance in fear conditioning test (Fig. 7d–g). These results establish a direct causal relationship of PirB proteolysis with Golgi dysfunction and cognitive decline in AD.

### Discussion

Our study establishes proteolytic receptor processing as a critical link between neuroimmune signaling and organelle dysfunction in AD. Through systematic proteomic screening of human CSF sample, we identified PirB, an AD risk gene product, as a substrate for Aβ oligomer-triggered MMP cleavage. In contrast to previous studies that have predominantly focused on amyloidogenic targets like APP, our study identified non-amyloidogenic membrane receptors that mediate key processes, including neuroimmune crosstalk. These findings offer a mechanistic bridge between immune receptor activation and proteostasis failure in early AD. Furthermore, these findings link proteolytic receptor processing to vesicular trafficking deficits and cognitive decline in AD, highlighting the C-terminal fragment of the immune receptor as an autonomous intracellular signal transducer. The elevated PirB-CTF combined with Aβ impairs the functions of Golgi apparatus and mediates neuronal degeneration. Our study suggests a shared regulatory node between neuroimmune receptors (like PirB) and classic amyloidogenic pathways.

In AD, upon ligand (like Notch1)-dependent cleavage [55], the level of PirB-CTF is persistently elevated, reflecting a dysregulation of proteostasis due to chronic Aβ oligomer binding. Furthermore, many potential ligands may also enhance the cleavage of PirB. A recent study showed that C4d can bind to PirB at the same binding site as Aβ oligomer [36]. Here we also found that C4d enhanced the cleavage of PirB. The expression level of MHC-I gradually decreases during development, a pattern similar to that of PirB-CTF (data not shown). This suggests that MHC-I may also regulate the cleavage of PirB [56]. Identifying ligands that can regulate the cleavage of PirB is critical for understanding how PirB functions in different environments.

PirB receptor mainly locates at peri-synaptic sites and axons [20, 34], and engages cytoplasmic phosphatases (SHP2/PP2A) at synapses [13, 20, 57]. Here, we found that the Aβ-caused cleavage product PirB-CTF is transported retrogradely via endosomes to the trans-Golgi apparatus, where GGA3 is sequestered via its GAT domain. GGA3 plays a key role in mediating the retrograde trafficking of BACE1 and other AD-related cargos, regulating neuronal synapses and signal transmission [49, 58]. The impairment of GGA3-dependent sorting caused by PirB-CTF indicates an association between two AD hallmarks via receptor cleavage: lysosomal dysfunction (reduced CTSD maturation) and Aβ overproduction (via impaired BACE1 regulation). Previous studies have shown that dysfunction of lysosomes and excessive production of Aβ are mutually reinforcing. Aβ can target lysosomes and increase the permeability of lysosomal membranes, resulting in the leakage of the hydrolase from lysosomes. Meanwhile, the increased membrane permeability also causes defective lysosomal acidification, which reduces the activity of many key hydrolases, such as CTSD [59]. On the other hand, dysfunctional lysosomes are unable to degrade Aβ efficiently, which further aggravates the accumulation of Aβ [60]. Our study provides new insights into the mechanisms by which Aβ affects lysosomal function, integrating extracellular signals, Aβ receptors, Golgi apparatus, and lysosomes into a network.

Our identification of the PirB-CTF/GGA3 interaction as a druggable target addresses a critical gap in AD therapeutics, which is consistent with the evidence that *GGA3* variants are linked to late-onset AD [49, 58] and its depletion exacerbates Aβ pathology. However, our current intervention methods still have certain limitations. For instance, MMP inhibitors are easier to administer, but the lack of specificity limits its application. Although GAT domain is more specific, its delivery method is invasive. Therefore, searching for drugs that block the interaction between PirB and MMPs, inhibiting PirB-CTF endocytosis, and blocking the interactions between PirB-CTF and GGA3 would be future research directions.

A limitation of this study is the exclusive use of male mice. It has been clinically proven that there are sex differences in AD [61–63]. Women have a higher lifetime risk of developing AD and often exhibit a distinct pathological profile, including greater tau pathology and cognitive decline, even at comparable levels of amyloid burden. Due to the possible influence of estrogen and other sex hormones, the cleavage rate of PirB, the stability of PirB-CTF, or its interaction with GGA3 may differ between males and females. Therefore, whether the level of LILRB2 cleavage in patient brains and the function of PirB-CTF in the mouse brain are influenced by biological sex remains an important open question for future investigation.

Overall, our study highlights an unexpected role of receptor fragments as intracellular signal conductors. Specifically, we demonstrate that PirB-CTF, rather than its full-length counterpart, drives Golgi function impairment. This suggests a model in which proteolytic fragments switch physiological cleavage events (e.g., developmental pruning) into pathological cascades. Furthermore, in addition to the products of splicing of AD-related genes, there might be other immunoinhibitory receptors among the 842 screened membrane proteins that undergo cleavage and are involved in other diseases such as epilepsy, autism and neuropsychiatric disorders. Our work thus provides a unifying framework for understanding organelle dysfunction in neurodegeneration and opens an avenue for therapeutic intervention.

## Conclusions

In conclusion, our study elucidates that the immunoinhibitory receptor PirB connects the extracellular environment with intracellular proteotoxicity and organelle dysfunction in AD pathology. The ligand-dependent PirB cleavage impairs Golgi-associated protein transport function, resulting in impaired synaptic vesicle transport and lysosomal maturation. Blocking PirB-CTF cleavage or its function may provide a therapeutic strategy to maintain intracellular homeostasis in AD.

## Supplementary Information

**Additional file 1**. **Fig. S1** Analysis of putatively cleavage protein in human CSF and PirB undergo cleavage.** Fig. S2** Analysis of cleavage site, the preparation of sPirB and Aβ-oligo, and regulation of PirB cleavage. **Fig. S3** PirB-CTF targets to Golgi apparatus. **Fig. S4** Validation of shRNA and dynamic images of neurite outgrowth. **Fig. S5** PirB-CTF colocalizes with GGA3, not ARCN1, and GAT can reverse the neurite outgrowth inhibition caused by excessive PirB-CTF. **Fig. S6** Reducing PirB-CTF production by GM can reverse Golgi apparatus function in AD mice.

**Additional file 2.** **Movie S1.** Time lapse imaging of the synchronized trafficking of ssTdtomato in vehicle, PirB, PirB-mut and PirB-CTF transfected HEK293T cell.

**Additional file 3. Movie S2.** Time lapse imaging of the trafficking of NPY-mCherry in vehicle, PirB-FL, PirB-mut and PirB-CTF transfected PirB KO hippocampus neuron.

**Additional file 4. Movie S3.** Time lapse imaging of the synchronized trafficking of ssTdtomato in PirB-CTF transfected HEK293T cell, with or without GAT domain.

**Additional file 5. Movie S4.** Time lapse imaging of the trafficking of NPY-mCherry in vehicle and PirB-CTF transfected PirB KO hippocampus neuron, with or without GAT domain.

**Additional file 6.** **Movie S5.** Time lapse imaging of the trafficking of NPY-mCherry in DMSO and Aβ oligomer treated WT hippocampus neuron, with or without GAT domain.

** Additional file 7**. Original Western blots.

## Data Availability

All data generated or analysed during this study are included in this published article and its supplementary information files. Further inquiries can be made to the corresponding authors.

## Abbreviations

Aβ: Amyloid-β
AD: Alzheimer’s disease
APP: Amyloid precursor protein
BMT: Barnes maze test
CTF: C-terminal fragment
CTSD: Cathepsin D
ESCRT: Endosomal sorting complexes required for transport
GFAP: Glial fibrillary acidic protein
GGA3: Gamma-ear-containing ARF binding protein 3
GO: Gene ontology
Iba1: Ionized calcium binding adaptor molecule 1
LC–MS/MS: Liquid chromatography-tandem mass spectrometry
LILRB2: Leukocyte immunoglobulin like receptor B2
MMPs: Matrix metallopeptidases
NPY: Neuropeptide Y
NTF: N-terminal fragment
PirB: Paired immunoglobulin-like receptor B
PLA: Proximity ligation assay
RUSH: Retention using selective hooks
sPirB: Soluble PirB ectodomain
TGN46: Trans-golgi network protein 46

## References

[1] Sprinzak D, Blacklow SC. Biophysics of notch signaling. Annu Rev Biophys. 2021;50:157–89. 33534608 10.1146/annurev-biophys-101920-082204 PMC8105286

[2] Steiner A, Schlepckow K, Brunner B, Steiner H, Haass C, Hagn F. Gamma-secretase cleavage of the Alzheimer risk factor TREM2 is determined by its intrinsic structural dynamics. EMBO J. 2020;39(20):e104247. 32830336 10.15252/embj.2019104247 PMC7560206

[3] Zhang H, Ma Q, Zhang YW, Xu H. Proteolytic processing of Alzheimer’s beta-amyloid precursor protein. J Neurochem. 2012;120(Suppl 1(Suppl 1)):9–21. 22122372 10.1111/j.1471-4159.2011.07519.x PMC3254787

[4] Bray SJ. Notch signalling: a simple pathway becomes complex. Nat Rev Mol Cell Biol. 2006;7(9):678–89. 16921404 10.1038/nrm2009

[5] Muller UC, Deller T, Korte M. Not just amyloid: physiological functions of the amyloid precursor protein family. Nat Rev Neurosci. 2017;18(5):281–98. 28360418 10.1038/nrn.2017.29

[6] Conant K, Daniele S, Bozzelli PL, Abdi T, Edwards A, Szklarczyk A, et al. Matrix metalloproteinase activity stimulates N-cadherin shedding and the soluble N-cadherin ectodomain promotes classical microglial activation. J Neuroinflammation. 2017;14(1):56. 28302163 10.1186/s12974-017-0827-4 PMC5356362

[7] Filipello F, Goldsbury C, You SF, Locca A, Karch CM, Piccio L. Soluble TREM2: innocent bystander or active player in neurological diseases? Neurobiol Dis. 2022;165:105630. 35041990 10.1016/j.nbd.2022.105630 PMC10108835

[8] Servian-Morilla E, Robles-Lanuza E, Sanchez-Hidalgo AC, Camacho-Garcia RJ, Paez-Gomez JA, Mavillard F, et al. Proteolytic processing of neurexins by presenilins sustains synaptic vesicle release. J Neurosci. 2018;38(4):901–17. 29229705 10.1523/JNEUROSCI.1357-17.2017 PMC6596238

[9] Borggrefe T, Lauth M, Zwijsen A, Huylebroeck D, Oswald F, Giaimo BD. The notch intracellular domain integrates signals from Wnt, hedgehog, TGFbeta/BMP and hypoxia pathways. Biochim Biophys Acta. 2016;1863(2):303–13. 26592459 10.1016/j.bbamcr.2015.11.020

[10] Ennerfelt H, Holliday C, Shapiro DA, Zengeler KE, Bolte AC, Ulland TK, et al. CARD9 attenuates Abeta pathology and modifies microglial responses in an Alzheimer’s disease mouse model. Proc Natl Acad Sci USA. 2023;120(24):e2303760120. 37276426 10.1073/pnas.2303760120 PMC10268238

[11] Bu XL, Sun PY, Fan DY, Wang J, Sun HL, Cheng Y, et al. Associations of plasma soluble CD22 levels with brain amyloid burden and cognitive decline in Alzheimer’s disease. Sci Adv. 2022;8(13):eabm5667. 35363517 10.1126/sciadv.abm5667 PMC10938586

[12] Eskandari-Sedighi G, Jung J, Macauley MS. CD33 isoforms in microglia and Alzheimer’s disease: friend and foe. Mol Aspects Med. 2023;90:101111. 35940942 10.1016/j.mam.2022.101111

[13] Kim T, Vidal GS, Djurisic M, William CM, Birnbaum ME, Garcia KC, et al. Human LilrB2 is a beta-amyloid receptor and its murine homolog PirB regulates synaptic plasticity in an Alzheimer’s model. Science. 2013;341(6152):1399–404. 24052308 10.1126/science.1242077 PMC3853120

[14] Rumpret M, Drylewicz J, Ackermans LJE, Borghans JAM, Medzhitov R, Meyaard L. Functional categories of immune inhibitory receptors. Nat Rev Immunol. 2020;20(12):771–80. 32612208 10.1038/s41577-020-0352-z

[15] Steevels TA, Meyaard L. Immune inhibitory receptors: essential regulators of phagocyte function. Eur J Immunol. 2011;41(3):575–87. 21312193 10.1002/eji.201041179

[16] Navarro-Alvarez N, Yang YG. CD47: a new player in phagocytosis and xenograft rejection. Cell Mol Immunol. 2011;8(4):285–8. 21258362 10.1038/cmi.2010.83 PMC3644051

[17] Samuels JD, Lukens JR, Price RJ. Emerging roles for ITAM and ITIM receptor signaling in microglial biology and Alzheimer’s disease-related amyloidosis. J Neurochem. 2024;168(10):3558–73. 37822118 10.1111/jnc.15981 PMC11955997

[18] Linnartz B, Neumann H. Microglial activatory (immunoreceptor tyrosine-based activation motif)- and inhibitory (immunoreceptor tyrosine-based inhibition motif)-signaling receptors for recognition of the neuronal glycocalyx. Glia. 2013;61(1):37–46. 22615186 10.1002/glia.22359

[19] Syken J, Grandpre T, Kanold PO, Shatz CJ. PirB restricts ocular-dominance plasticity in visual cortex. Science. 2006;313(5794):1795–800. 16917027 10.1126/science.1128232

[20] Djurisic M, Brott BK, Saw NL, Shamloo M, Shatz CJ. Activity-dependent modulation of hippocampal synaptic plasticity via PirB and endocannabinoids. Mol Psychiatry. 2019;24(8):1206–19. 29670176 10.1038/s41380-018-0034-4 PMC6372352

[21] Ujike A, Takeda K, Nakamura A, Ebihara S, Akiyama K, Takai T. Impaired dendritic cell maturation and increased T(H)2 responses in PIR-B(-/-) mice. Nat Immunol. 2002;3(6):542–8. 12021780 10.1038/ni801

[22] Fan X, Shi P, Dai J, Lu Y, Chen X, Liu X, et al. Paired immunoglobulin-like receptor B regulates platelet activation. Blood. 2014;124(15):2421–30. 25075127 10.1182/blood-2014-03-557645 PMC4192752

[23] Zheng J, Umikawa M, Cui C, Li J, Chen X, Zhang C, et al. Inhibitory receptors bind ANGPTLs and support blood stem cells and leukaemia development. Nature. 2012;485(7400):656–60. 22660330 10.1038/nature11095 PMC3367397

[24] Sun S, Zhang H, Liu J, Popugaeva E, Xu NJ, Feske S, et al. Reduced synaptic STIM2 expression and impaired store-operated calcium entry cause destabilization of mature spines in mutant presenilin mice. Neuron. 2014;82(1):79–93. 24698269 10.1016/j.neuron.2014.02.019 PMC4007018

[25] Liu XD, Zhu XN, Halford MM, Xu TL, Henkemeyer M, Xu NJ. Retrograde regulation of mossy fiber axon targeting and terminal maturation via postsynaptic Lnx1. J Cell Biol. 2018;217(11):4007–24. 30185604 10.1083/jcb.201803105 PMC6219728

[26] Park K, Lee J, Jang HJ, Richards BA, Kohl MM, Kwag J. Optogenetic activation of parvalbumin and somatostatin interneurons selectively restores theta-nested gamma oscillations and oscillation-induced spike timing-dependent long-term potentiation impaired by amyloid beta oligomers. BMC Biol. 2020;18(1):7. 31937327 10.1186/s12915-019-0732-7 PMC6961381

[27] Boncompain G, Divoux S, Gareil N, de Forges H, Lescure A, Latreche L, et al. Synchronization of secretory protein traffic in populations of cells. Nat Methods. 2012;9(5):493–8. 22406856 10.1038/nmeth.1928

[28] Dammer EB, Ping L, Duong DM, Modeste ES, Seyfried NT, Lah JJ, et al. Multi-platform proteomic analysis of Alzheimer’s disease cerebrospinal fluid and plasma reveals network biomarkers associated with proteostasis and the matrisome. Alzheimers Res Ther. 2022;14(1):174. 36384809 10.1186/s13195-022-01113-5 PMC9670630

[29] Wightman DP, Jansen IE, Savage JE, Shadrin AA, Bahrami S, Holland D, et al. A genome-wide association study with 1,126,563 individuals identifies new risk loci for Alzheimer’s disease. Nat Genet. 2021;53(9):1276–82. 34493870 10.1038/s41588-021-00921-z PMC10243600

[30] Bellenguez C, Kucukali F, Jansen IE, Kleineidam L, Moreno-Grau S, Amin N, et al. New insights into the genetic etiology of Alzheimer’s disease and related dementias. Nat Genet. 2022;54(4):412–36. 35379992 10.1038/s41588-022-01024-z PMC9005347

[31] Kunkle BW, Grenier-Boley B, Sims R, Bis JC, Damotte V, Naj AC, et al. Genetic meta-analysis of diagnosed Alzheimer’s disease identifies new risk loci and implicates Abeta, tau, immunity and lipid processing. Nat Genet. 2019;51(3):414–30. 30820047 10.1038/s41588-019-0358-2 PMC6463297

[32] Jansen IE, Savage JE, Watanabe K, Bryois J, Williams DM, Steinberg S, et al. Genome-wide meta-analysis identifies new loci and functional pathways influencing Alzheimer’s disease risk. Nat Genet. 2019;51(3):404–13. 30617256 10.1038/s41588-018-0311-9 PMC6836675

[33] Lambert JC, Ibrahim-Verbaas CA, Harold D, Naj AC, Sims R, Bellenguez C, et al. Meta-analysis of 74,046 individuals identifies 11 new susceptibility loci for Alzheimer’s disease. Nat Genet. 2013;45(12):1452–8. 24162737 10.1038/ng.2802 PMC3896259

[34] Adelson JD, Barreto GE, Xu L, Kim T, Brott BK, Ouyang YB, et al. Neuroprotection from stroke in the absence of MHCI or PirB. Neuron. 2012;73(6):1100–7. 22445338 10.1016/j.neuron.2012.01.020 PMC3314229

[35] Bochner DN, Sapp RW, Adelson JD, Zhang S, Lee H, Djurisic M, et al. Blocking PirB up-regulates spines and functional synapses to unlock visual cortical plasticity and facilitate recovery from amblyopia. Sci Transl Med. 2014;6(258):258ra140. 25320232 10.1126/scitranslmed.3010157 PMC4476552

[36] Brott BK, Raissi AJ, Micheva KD, Vielmetter J, Mendes MS, Baccus CJ, et al. C4d, a high-affinity LilrB2 ligand, is elevated in Alzheimer’s disease and mediates synapse pruning. Proc Natl Acad Sci USA. 2025;122(38):e2519253122. 40966293 10.1073/pnas.2519253122 PMC12478167

[37] Das U, Wang L, Ganguly A, Saikia JM, Wagner SL, Koo EH, et al. Visualizing APP and BACE-1 approximation in neurons yields insight into the amyloidogenic pathway. Nat Neurosci. 2016;19(1):55–64. 26642089 10.1038/nn.4188 PMC4782935

[38] Choy RW, Cheng Z, Schekman R. Amyloid precursor protein (APP) traffics from the cell surface via endosomes for amyloid beta (Abeta) production in the trans-Golgi network. Proc Natl Acad Sci USA. 2012;109(30):E2077–82. 22711829 10.1073/pnas.1208635109 PMC3409748

[39] He J, Zhang L. The journey of STING: guiding immune signaling through membrane trafficking. Cytokine Growth Factor Rev. 2024;78:25–36. 39019665 10.1016/j.cytogfr.2024.07.003

[40] Pei Y, Lv S, Shi Y, Jia J, Ma M, Han H, et al. RAB21 controls autophagy and cellular energy homeostasis by regulating retromer-mediated recycling of SLC2A1/GLUT1. Autophagy. 2023;19(4):1070–86. 35993307 10.1080/15548627.2022.2114271 PMC10012929

[41] Williams ET, Chen X, Otero PA, Moore DJ. Understanding the contributions of VPS35 and the retromer in neurodegenerative disease. Neurobiol Dis. 2022;170:105768. 35588987 10.1016/j.nbd.2022.105768 PMC9233057

[42] MacDonald E, Brown L, Selvais A, Liu H, Waring T, Newman D, et al. HRS-WASH axis governs actin-mediated endosomal recycling and cell invasion. J Cell Biol. 2018;217(7):2549–64. 29891722 10.1083/jcb.201710051 PMC6028553

[43] Vazquez-Sanchez S, Bobeldijk S, Dekker MP, van Keimpema L, van Weering JRT. VPS35 depletion does not impair presynaptic structure and function. Sci Rep. 2018;8(1):2996. 29445238 10.1038/s41598-018-20448-4 PMC5812998

[44] Luo Q, Liu Q, Cheng H, Wang J, Zhao T, Zhang J, et al. Nondegradable ubiquitinated ATG9A organizes Golgi integrity and dynamics upon stresses. Cell Rep. 2022;40(7):111195. 35977480 10.1016/j.celrep.2022.111195

[45] Joshi G, Chi Y, Huang Z, Wang Y. Abeta-induced Golgi fragmentation in Alzheimer’s disease enhances Abeta production. Proc Natl Acad Sci USA. 2014;111(13):E1230–9. 24639524 10.1073/pnas.1320192111 PMC3977293

[46] Nakazawa H, Sada T, Toriyama M, Tago K, Sugiura T, Fukuda M, et al. Rab33a mediates anterograde vesicular transport for membrane exocytosis and axon outgrowth. J Neurosci. 2012;32(37):12712–25. 22972995 10.1523/JNEUROSCI.0989-12.2012 PMC6703789

[47] Zhang Y, Kunii M, Taniguchi M, Yoshimura SI, Harada A. Rab6-mediated polarized transport of synaptic vesicle precursors is essential for the establishment of neuronal polarity and brain formation. J Neurosci. 2024. 10.1523/JNEUROSCI.2334-23.2024. 38830762 10.1523/JNEUROSCI.2334-23.2024 PMC11223463

[48] Guedes-Dias P, Holzbaur ELF. Axonal transport: driving synaptic function. Science. 2019. 10.1126/science.aaw9997. 31601744 10.1126/science.aaw9997 PMC6996143

[49] Lomoio S, Willen R, Kim W, Ho KZ, Robinson EK, Prokopenko D, et al. Gga3 deletion and a GGA3 rare variant associated with late onset Alzheimer’s disease trigger BACE1 accumulation in axonal swellings. Sci Transl Med. 2020. 10.1126/scitranslmed.aba1871. 33208500 10.1126/scitranslmed.aba1871 PMC8612295

[50] Santos TC, Wierda K, Broeke JH, Toonen RF, Verhage M. Early Golgi abnormalities and neurodegeneration upon loss of presynaptic proteins Munc18-1, Syntaxin-1, or SNAP-25. J Neurosci. 2017;37(17):4525–39. 28348137 10.1523/JNEUROSCI.3352-16.2017 PMC6596660

[51] Shen Y, Zhao H, Li P, Peng Y, Cui P, Miao F, et al. MHC class I molecules and PirB shape neuronal morphology by affecting the dendritic arborization of cortical neurons. Neurochem Res. 2019;44(2):312–22. 30406910 10.1007/s11064-018-2676-7

[52] Maeda A, Kurosaki M, Ono M, Takai T, Kurosaki T. Requirement of SH2-containing protein tyrosine phosphatases SHP-1 and SHP-2 for paired immunoglobulin-like receptor B (PIR-B)-mediated inhibitory signal. J Exp Med. 1998;187(8):1355–60. 9547347 10.1084/jem.187.8.1355 PMC2212224

[53] Bloom GS. Amyloid-beta and tau: the trigger and bullet in Alzheimer disease pathogenesis. JAMA Neurol. 2014;71(4):505–8. 24493463 10.1001/jamaneurol.2013.5847 PMC12908160

[54] Li Y, Qi J, Guo L, Jiang X, He G. Organellar quality control crosstalk in aging-related disease: innovation to pave the way. Aging Cell. 2025;24(1):e14447. 39668579 10.1111/acel.14447 PMC11709098

[55] Zhou B, Lin W, Long Y, Yang Y, Zhang H, Wu K, et al. Notch signaling pathway: architecture, disease, and therapeutics. Signal Transduct Target Ther. 2022;7(1):95. 35332121 10.1038/s41392-022-00934-y PMC8948217

[56] Needleman LA, Liu XB, El-Sabeawy F, Jones EG, McAllister AK. MHC class I molecules are present both pre- and postsynaptically in the visual cortex during postnatal development and in adulthood. Proc Natl Acad Sci USA. 2010;107(39):16999–7004. 20837535 10.1073/pnas.1006087107 PMC2947898

[57] Meng X, Li D, Kan R, Xiang Y, Pan L, Guo Y, et al. Inhibition of ANGPTL8 protects against diabetes-associated cognitive dysfunction by reducing synaptic loss via the PirB signaling pathway. J Neuroinflammation. 2024;21(1):192. 39095838 10.1186/s12974-024-03183-8 PMC11297729

[58] Kim W, Ma L, Lomoio S, Willen R, Lombardo S, Dong J, et al. BACE1 elevation engendered by GGA3 deletion increases beta-amyloid pathology in association with APP elevation and decreased CHL1 processing in 5XFAD mice. Mol Neurodegener. 2018;13(1):6. 29391027 10.1186/s13024-018-0239-7 PMC5796504

[59] Colacurcio DJ, Pensalfini A, Jiang Y, Nixon RA. Dysfunction of autophagy and endosomal-lysosomal pathways: roles in pathogenesis of Down syndrome and Alzheimer’s disease. Free Radic Biol Med. 2018;114:40–51. 28988799 10.1016/j.freeradbiomed.2017.10.001 PMC5748263

[60] Nixon RA. Autophagy-lysosomal-associated neuronal death in neurodegenerative disease. Acta Neuropathol. 2024;148(1):42. 39259382 10.1007/s00401-024-02799-7 PMC11418399

[61] Kolahchi Z, Henkel N, Eladawi MA, Villarreal EC, Kandimalla P, Lundh A, et al. Sex and gender differences in Alzheimer’s disease: genetic, hormonal, and inflammation impacts. Int J Mol Sci. 2024. 10.3390/ijms25158485. 39126053 10.3390/ijms25158485 PMC11313277

[62] Acosta-Martinez M, Carter V, Nessim A, Panchal R, John D, Dhawan J, et al. Sex- and region-dependent neuroinflammation in Alzheimer’s disease. Alzheimers Dement. 2025;21(4):e14603. 40247152 10.1002/alz.14603 PMC12005983

[63] Li A, Klinger HM, Seto M, Birkenbihl C, Properzi MJ, Farrell M, et al. Elevated temporal tau PET predicts faster cognitive decline in women than men: a meta-analysis. Alzheimers Dement. 2026;22(2):e71031. 41705602 10.1002/alz.71031 PMC12914651

