# Supplementary material for: Persistent PirB cleavage drives Golgi-directed trafficking deficits underlying neurodegeneration
Source: Transl Neurodegener. 2026 Jun 3;15:26. doi: 10.1186/s40035-026-00553-5 (PMC13231542; doi:10.1186/s40035-026-00553-5)
Supplement: Supplementary file 1 — Additional file 1. Fig. S1 Analysis of putatively cleavage protein in human CSF and PirB undergo cleavage. Fig. S2 Analysis of cleavage site, the preparation of sPirB and Aβ-oligo, and regulation of PirB cleavage. Fig. S3 PirB-CTF targets to Golgi apparatus. Fig. S4 Validation of shRNA and dynamic images of neurite outgrowth. Fig. S5 PirB-CTF colocalizes with GGA3, not ARCN1, and GAT can reverse the neurite outgrowth inhibition caused by excessive PirB-CTF. Fig. S6 Reducing PirB-CTF production by GM can reverse Golgi apparatus function in AD mice. [file 40035_2026_553_MOESM1_ESM.pdf]

Persistent PirB cleavage drives Golgi-directed trafficking deficits underlying neurodegeneration

Wu-Bo Han, Xian-Dong Liu, Chang-Fei Tang, Junke Zheng, Tian-Le Xu, Nan-Jie Xu*, Suya Sun*

*Corresponding Author:
Suya Sun, Ph.D. Email: sunsuya@shsmu.edu.cn;
Nan-Jie Xu, Ph.D. Email: xunanjie@sjtu.edu.cn

Supporting Information includes:

Figure S1 to S6

Legends for movies S1 to S5

Supplementary Figures

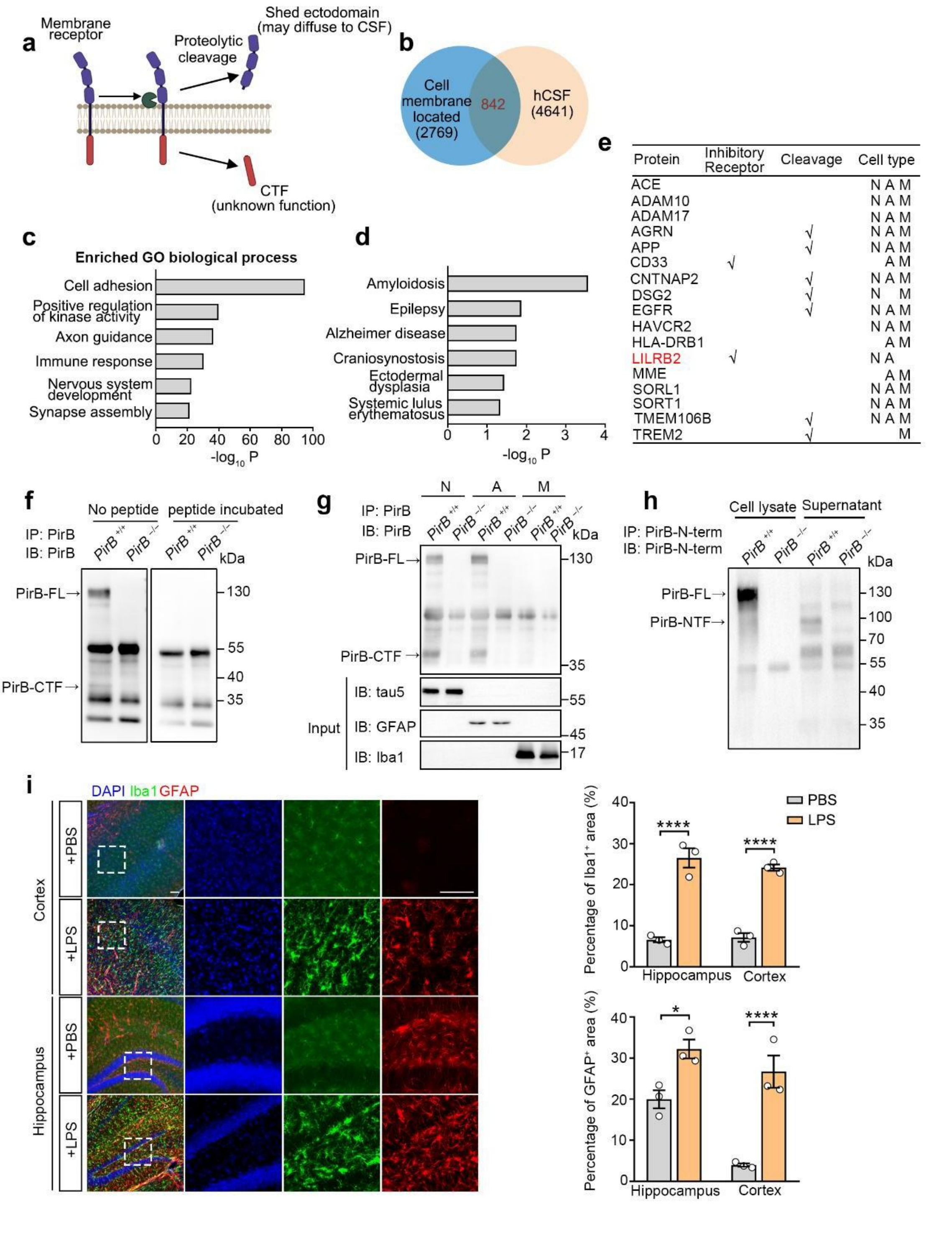

Fig. S1 Analysis of putatively cleavage protein in human CSF and PirB undergo cleavage. a, Schematic of the strategy for screening out the proteins which undergo proteolytic cleavage in CNS. **b,** Venn diagram showing that 842 (membrane-anchored) of 4641 proteins detects in human CSF may undergo proteolytic cleavage. **c,** GO analysis of 842 membrane-anchored proteins in human CSF. **d**, Enrichment of epilepsy and AD-related genes in the membrane-anchored subset of human CSF. **e**, Basic information of 17 proteins related to AD, which undergo proteolytic cleavage. N, neuron; A, astrocyte; M, microglia. **f,** Immunoprecipitation samples from the hippocampus of adult WT and *PirB* KO mouse were analyzed by immunoblot analysis using anti-PirB. The antibody recognized bands at ~130 (PirB-FL) and ~40 kDa (PirB-CTF).

Immunoreactivity was completely blocked when antibody was preincubated with 90 μM of the neutralized peptide. **g**, Expressions of PirB-FL and PirB-CTF in primary cultured neuron (Tau5^{+}), astrocyte (GFAP^{+}) and microglia (Iba1^{+}) are detected. **h**, N-terminal fragment of PirB (PirB-NTF) in intracellular lysate and extracellular supernatant was immunoprecipitated using anti-PirB-ecto. **i,** The verification of LPS injection in lateral ventricle. After drug administration, significant glial activation is observed in cortex and hippocampus (n = 3 mice per group, two-way ANOVA with Turkey's post hoc, *$P < 0.05$, ****$P < 0.001$).

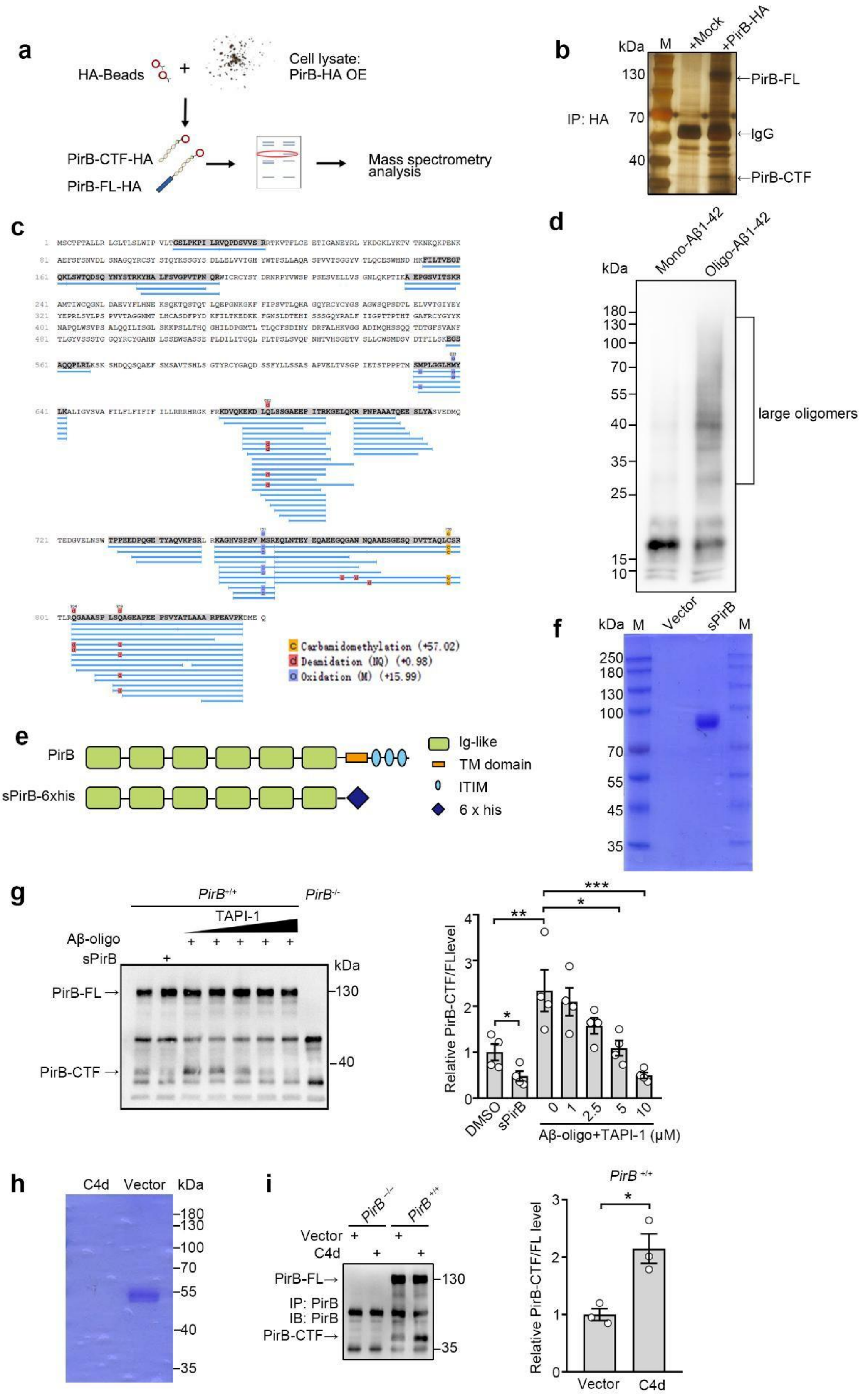

Fig. S2 Analysis of cleavage site, the preparation of sPirB and Aβ-oligo, and regulation of PirB cleavage. a, Flowchart of PirB proteolytic cleavage site exploration

experiment. **b**, Pattern of silver stained SDS-PAGE shows PirB-FL-HA and PirB-CTF-HA in cell lysate isolated by immunoprecipitation. **c**, Coverage of peptide detected by LC-MS/MS shows the cleavage occurred in extracellular juxtamembrane region. **d**, Validation of oligomeric Aβ by western blotting with 6E10 antibody. **e**, Schematic of soluble PirB-6xhis (sPirB) fusion protein showing extracellular Ig-like domain with His tag. **f**, Coomassie brilliant blue staining of sPirB. **g**, Endogenous PirB cleavage is prevented by ligand binding block (n = 4 biological replicates in each group, DMSO versus sPirB, unpaired t-test, *$P < 0.05$); and TAPI-1 decreases PirB cleavage upon Aβ oligomer stimulation (n = 4 biological replicates in each group, one-way ANOVA with Dunn's post hoc, *$P < 0.05$, **$P < 0.01$, ***$P < 0.001$). **h**, Coomassie brilliant blue staining of C4d. **i**, Detection of the effect of C4d on the cleavage of PirB in primary cultured hippocampal neurons. (n = 3 biological replicates in each group, unpaired t-test, *$P < 0.05$).

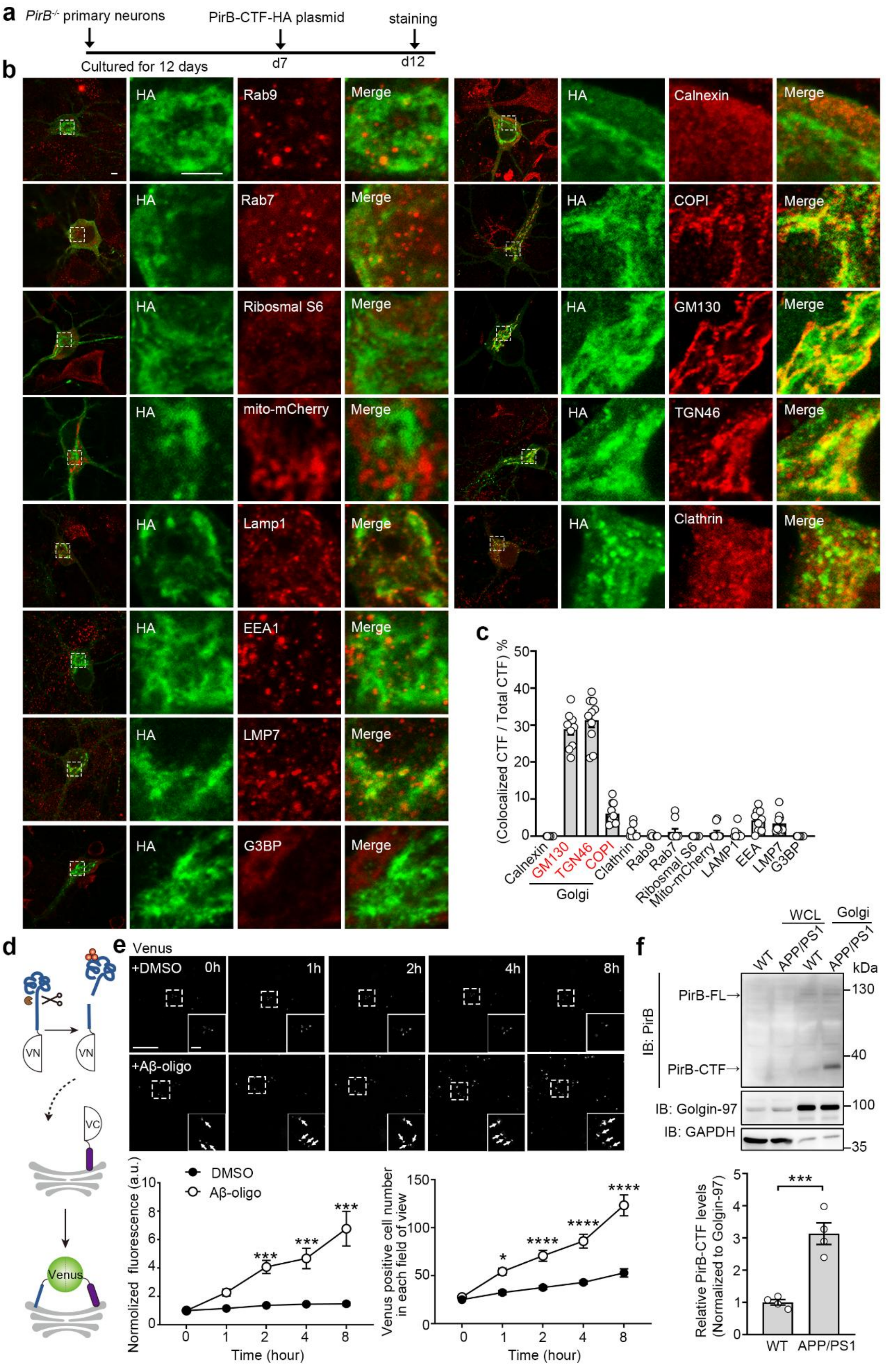

Fig. S3 PirB-CTF targets to Golgi apparatus. a, Flowchart of PirB-CTF-HA colocalization screen. HA-tagged (C-term) PirB-CTF was exogenously overexpressed in *PirB* KO hippocampus neurons. **b**, Single-plane confocal imaging displays the subcellular location of PirB-CTF-HA in hippocampal neurons. Rab9 and Rab7, late endosome; Ribosmal S6, Ribosome; mito-mcherry, mitochondria; LAMP1, lysosome; EEA1, early endosome; LMP7,

proteasome; G3BP, Stress granules; Calnexin, Endoplasmic reticulum; COPI, coatomer; GM130, cis-Golgi apparatus; TGN46, trans-Golgi apparatus; Clathrin, Clathrin-coated vesicles. Scale bar: 5 μm. **c**, Analysis of colocalization of PirB-CTF-HA signal and various organelle signals. The ratio of colocalized area to the total PirB-CTF-HA area is used as colocalized percentage. **d**, Schematic of fluorescence complement experiment. **e**, Validation of fluorescence complementation in HEK293T cell. Scale bar: 100 μm, 20 μm for magnified images. (for left statistical diagram, n = 28 cells from 4 biological replicates per group, two-way ANOVA with Sidak's post hoc, ***$P < 0.001$; for right statistical diagram, n = 4 views per group, two-way ANOVA with Sidak's post hoc, *$P < 0.05$, ****$P < 0.0001$). **f**, Level of PirB-CTF in whole tissue lysate and isolated Golgi apparatus from 9-month WT and APP/PS1 mice. The level of PirB-CTF is normalized to Golgi marker Golgin-97 (n = 4 mice, unpaired t-test, ***$P < 0.001$).

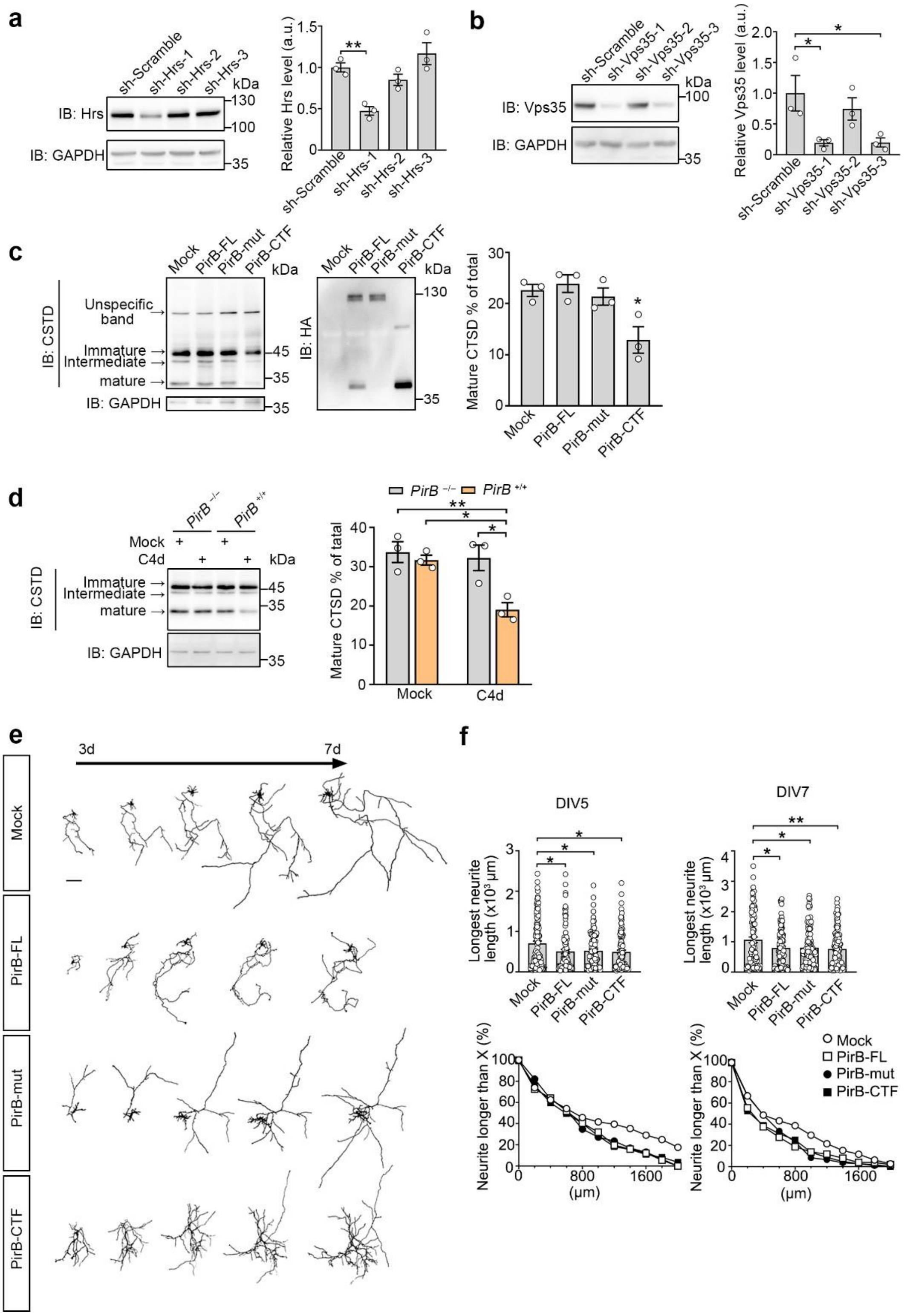

Fig. S4 Validation of shRNA and dynamic images of neurite outgrowth. a, Validation of shRNA of Hrs (n = 3 biological replicates per group, one-way ANOVA with Dunnett's post hoc, $^{**}P < 0.01$). **b**, Validation of shRNA of Vps35 (n = 3 biological replicates per group, one-way ANOVA with Dunnett's post hoc, $^{*}P < 0.05$). **c**, Measurement of maturation of CTSD in primary cultured *PirB* KO hippocampal neurons transfected with empty vector, PirB-FL, PirB-mut and PirB-CTF (with HA tag) for 72h (n = 3 biological replicates per group, one-way ANOVA with Sidak's post hoc, $^{*}P < 0.05$). **d**, Detection of the effect of C4d on the maturation of CTSD in *PirB* KO and WT primary cultured hippocampal neurons (n = 3 biological replicates per group, two-way ANOVA with Turkey's post hoc, $^{*}P < 0.05$, $^{**}P < 0.01$). **e**, Representative images of the dynamic

neurite outgrowth process of *PirB* KO hippocampal neurons transfected with empty vector, PirB-FL, PirB-mut or PirB-CTF. Scale bar: 100 µm. **f**, Comparison for the length of the longest neurites of hippocampal neurons transfected with empty vector, PirB-FL, PirB-mut or PirB-CTF (in DIV5 subset, n = 101-111 neurons from 5 biological replicates per group, one-way ANOVA with Turkey's post hoc, *$P < 0.05$; in DIV7 subset, n = 96-109 neurons from 5 biological replicates per group, one-way ANOVA with Turkey's post hoc, *$P < 0.05$, **$P < 0.01$).

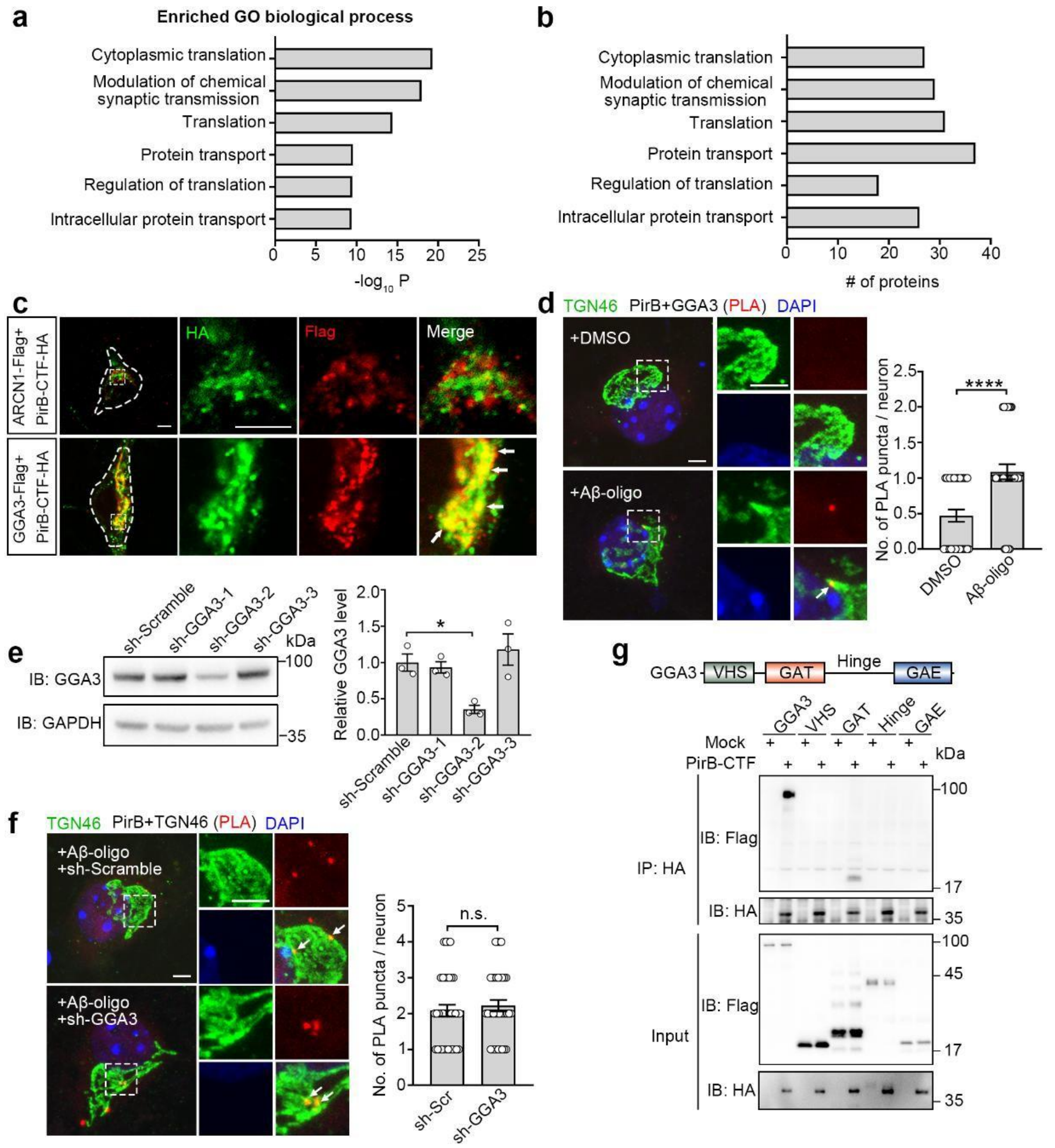

Fig. S5 PirB-CTF colocalizes with GGA3, not ARCN1, and GAT can reverse the neurite outgrowth inhibition caused by excessive PirB-CTF. a, GO analysis of PirB-CTF binding proteins by LC-MS/MS. The protein transport is highly enriched. **b**, Number of proteins of biological process. The number of protein transport process is highest. **c**, Co-localization of PirB-CTF-HA with ARCN1-Flag or GGA3-Flag in neurons. Arrows indicated colocalization. Scale bar: 5 µm. **d**, Confocal images of PLA puncta of WT neurons with the addition of DMSO or Aβ oligomers ($n = 34$ neurons from 3 biological replicates per group, unpaired t-test, $****P < 0.0001$). Scale bar: 5 µm. **e**, Validation of shRNA of GGA3 ($n = 3$ biological replicates per group, one-way ANOVA with Dunnett's post hoc, $*P < 0.05$). **f**, Confocal images of PLA puncta of Aβ oligomers treated WT neurons with transfection of empty or GGA3 shRNA ($n = 35$ neurons from 3 biological replicates per group, Unpaired t test). Scale bar: 5 µm. **g**, Co-IP of exogenously expressed PirB-CTF-HA and Flag tagged domains of GGA3 in HEK293T cells.

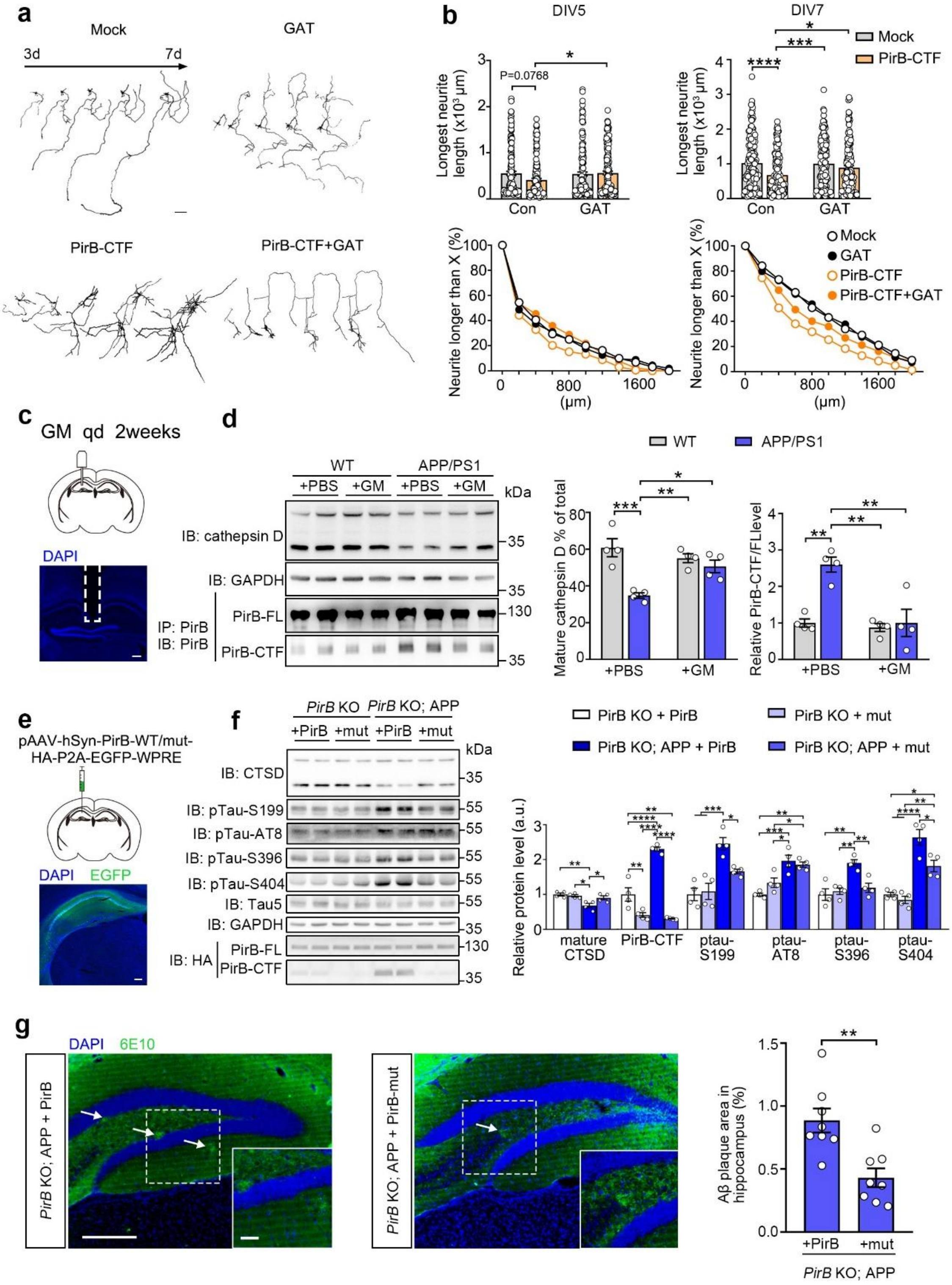

Fig. S6 Reducing PirB-CTF production by GM can reverse Golgi apparatus function in AD mice. a, Images of dynamic neurite outgrowth process of neurons transfected with empty vector or PirB-CTF in the presence or absence of GAT domain. Scale bar: 100 μm. **b**, Comparison for the longest length of the neurites of neurons transfected with empty vector or PirB-CTF in the presence or absence of GAT domain

(in DIV5 subset, *n* = 158-170 neurons from 5 biological replicates per group, two-way ANOVA with Sidak's post hoc, **P* < 0.05; in DIV7 subset, n=141-165 neurons from 5 biological replicates per group, two-way ANOVA with Sidak's post hoc, **P* < 0.05, ****P* < 0.001, *****P* < 0.0001). **c**, Experimental scheme of drug administration (1µg) in 6 months WT or APP/PS1 mouse hippocampus and histologically verified placements of drug catheter in hippocampus. The dash line indicated the outline of the catheter. Scale bar: 200 µm. **d**, Western bolting analysis of maturation of CTSD in WT or APP/PS1 mouse hippocampus in the presence or absence of GM6001. Bottom panel: Detection of PirB and PirB-CTF expression by immunoprecipitation (*n* = 4 mice, two-way ANOVA with Sidak's post hoc, **P* < 0.05, ***P* < 0.01, ****P* < 0.001). **e**, Experimental scheme of virus injection in 6 months WT or APP/PS1 mouse hippocampus and histologically verified placements of injection in hippocampus. Scale bar: 200 µm. **f**, Western bolting analysis of mature CTSD, pTau and PirB-CTF in WT or APP/PS1 (*PirB* KO background) mouse hippocampus in the presence of PirB or PirB-mut (*n* = 4 mice per group, two-way ANOVA with Tukey's post hoc, **P* < 0.05, ***P* < 0.01, ****P* < 0.001, *****P* < 0.0001). **g**, Immunofluorescence image of 6E10 staining in the brain section of 6-month-old *PirB*$^{-/-}$; APP/PS1 mice injected with PirB or PirB-mut virus. White arrows denote Aβ plaque, and dash squares denote the enlarged views. Scale bar: 200 µm, 50 µm for magnified images. The right panel shows the quantification of Aβ plaque area (*n*=8 mice per group, unpaired t-test, **P* < 0.05).

Supplementary Movie legends

Movie S1. Time lapse imaging of the synchronized trafficking of ssTdtomato in vehicle, PirB, PirB-mut and PirB-CTF transfected HEK293T cell.

KDEL-streptavidin (hook) and SBP-ssTdtomato (reporter) was co-expressed with vehicle, PirB-FL, PirB-mut and PirB-CTF in HEK293T cells. After 24 h of expression, biotin was added at 00:00 to induce the release of reporter. Scale bar: 10 μm.

Movie S2. Time lapse imaging of the trafficking of Npy-mCherry in vehicle, PirB-FL, PirB-mut and PirB-CTF transfected *PirB* KO hippocampus neuron.

Npy-mCherry was co-expressed with vehicle, PirB-FL, PirB-mut and PirB-CTF in primary *PirB* KO hippocampus neuron. After 48 h of expression, the trafficking of Npy-mCherry in axon segment region adjacent to soma was monitored. Scale bar: 10 μm.

Movie S3. Time lapse imaging of the synchronized trafficking of ssTdtomato in PirB-CTF transfected HEK293T cell, with or without GAT domain.

KDEL-streptavidin (hook) and SBP-ssTdtomato (reporter) was co-expressed with vehicle and PirB-CTF in HEK293T cells, in the presence or the absence of GAT domain. After 24 h of expression, biotin was added at 00:00 to induce the release of reporter. Scale bar: 10 μm.

Movie S4. Time lapse imaging of the trafficking of Npy-mCherry in vehicle and PirB-CTF transfected *PirB* KO hippocampus neuron, with or without GAT domain.

Npy-mCherry was co-expressed with vehicle and PirB-CTF in primary *PirB* KO hippocampus neuron, in the presence or the absence of GAT domain. After 48 h of expression, the trafficking of Npy-mCherry in axon segment region adjacent to soma was monitored. Scale bar: 10 μm.

Movie S5. Time lapse imaging of the trafficking of Npy-mCherry in DMSO and Aβ oligomer treated WT hippocampus neuron, with or without GAT domain.

Npy-mCherry was co-expressed with vehicle and GAT in primary wide-type hippocampus neuron. After 48 h of expression, the neurons were treated with DMSO or Aβ oligomer. After 4h treatment, the trafficking of Npy-mCherry in axon segment region adjacent to soma was monitored. Scale bar: 10 μm.
