# Supplementary material for: Persistent PirB cleavage drives Golgi-directed trafficking deficits underlying neurodegeneration
Source: Transl Neurodegener. 2026 Jun 3;15:26. doi: 10.1186/s40035-026-00553-5 (PMC13231542; doi:10.1186/s40035-026-00553-5)
Supplement: Supplementary file 7 — Additional file 7. Original Western blots. [file 40035_2026_553_MOESM7_ESM.pdf]

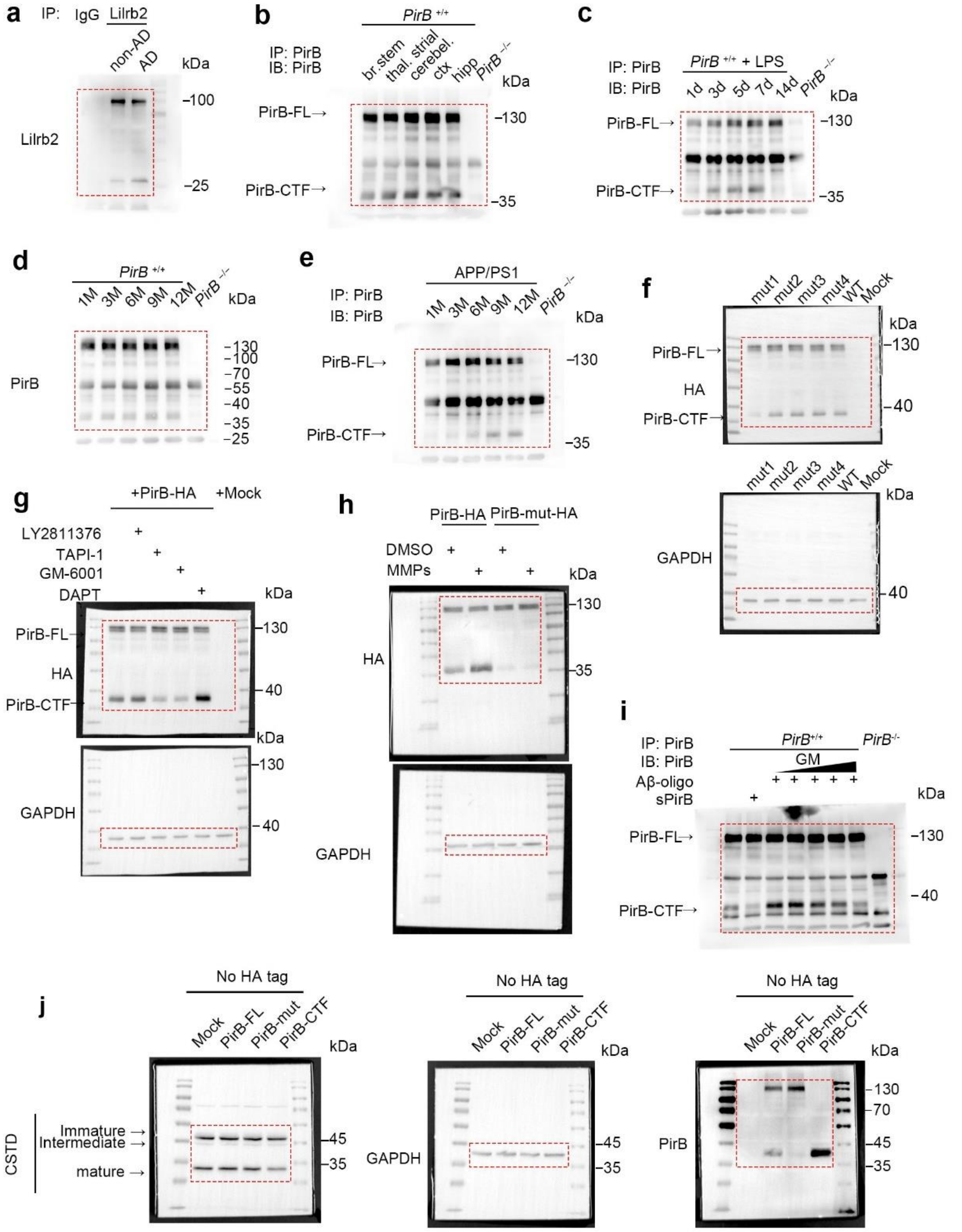

Uncut gels 1. The whole gel images for western blot results in Figure 1 and Figure 3. (**a**)**.** The whole gel image for Fig. 1c. (**b**). The whole gel image for Fig. 1d. (**c**). The whole gel image for Fig. 1e. (**d**). The whole gel image for Fig. 1f. (**e**). The whole gel image for Fig. 1g. (**f**). The whole gel image for Fig. 1i. (**g**). The whole gel image for Fig. 1j. (**h**). The whole gel image for Fig. 1k. (**i**) The whole gel image for Fig. 1l. (**j**) The whole gel image for Fig. 3b. Red dotted lines indicated the part showed in Figures.

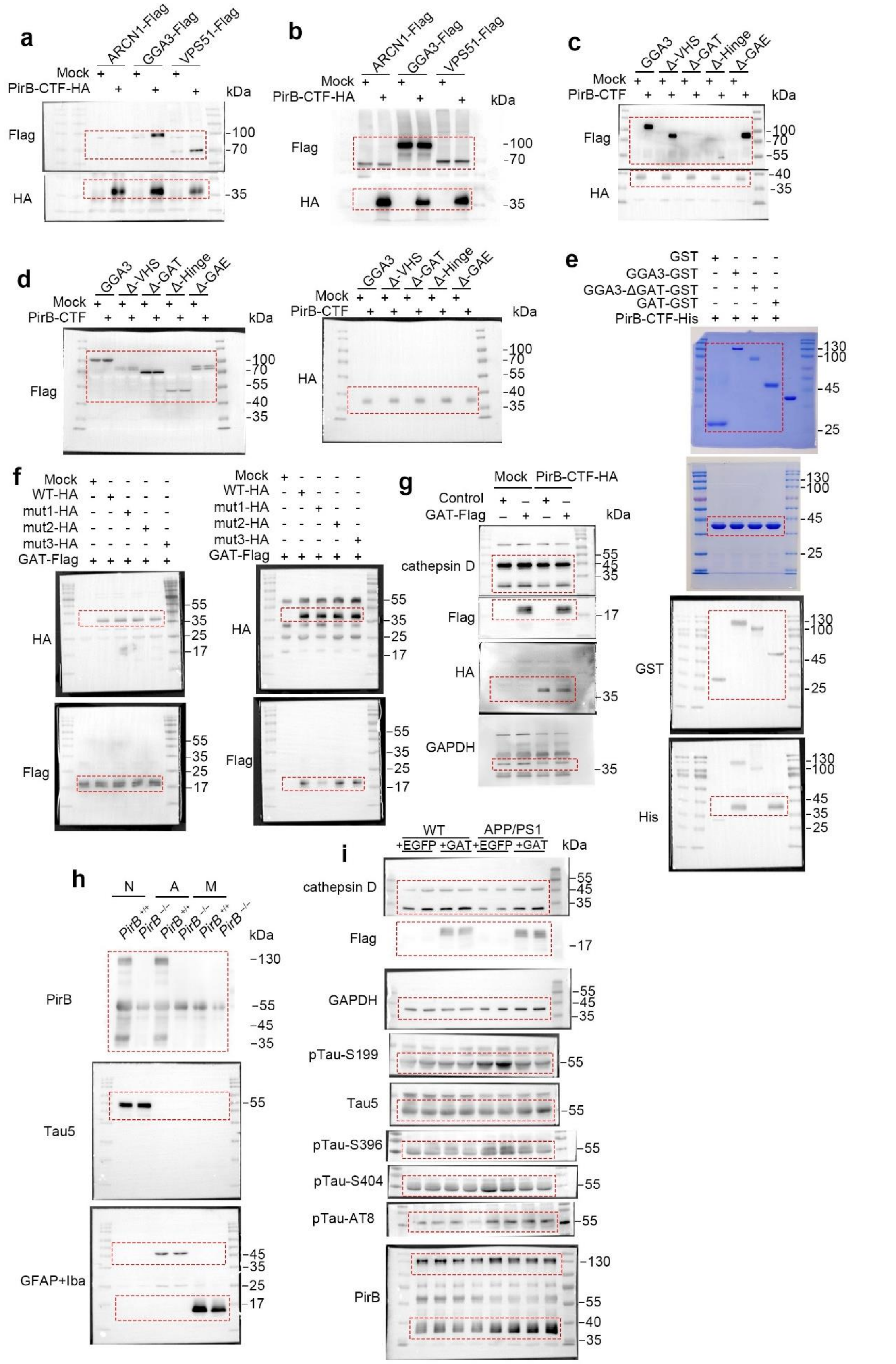

Uncut gels 2. The whole gel images for western blot results in Figure 4, Figure 5,

Figure 7 and Figure S1. (**a**)**.** The whole gel image for Fig. 4c (IP). (**b**)**.** The whole gel image for Fig. 4c (Input). (**c**)**.** The whole gel image for Fig. 4c (IP). (**d**)**.** The whole gel image for Fig. 4d (Input). (**e**)**.** The whole gel image for Fig. 4e. (**f**)**.** The whole gel image for Fig. 4f. (**g**)**.** The whole gel image for Fig. 5a. (**h**)**.** The whole gel image for Fig. S1g. (**i**)**.** The whole gel image for Fig. 6b.

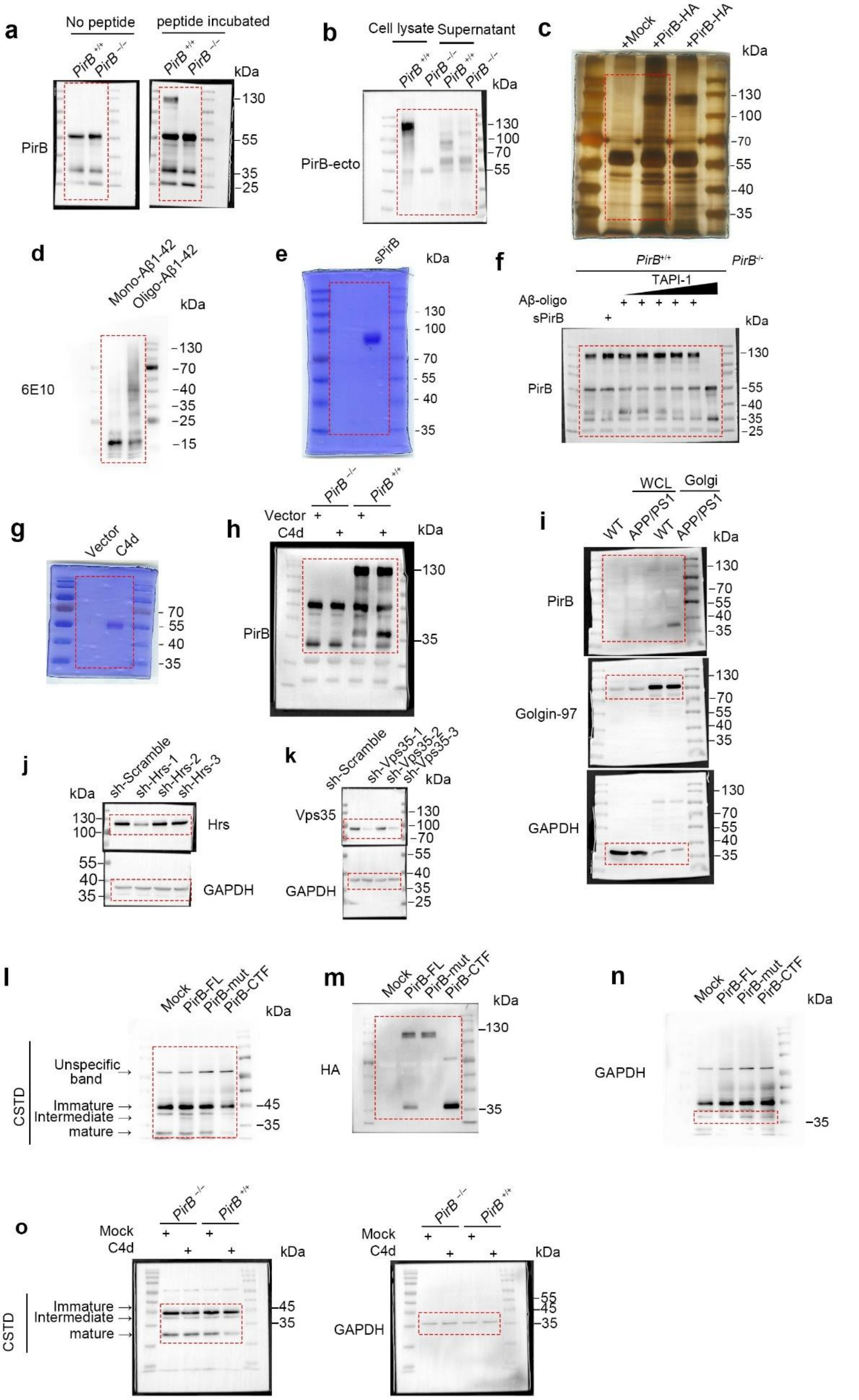

Uncut gels 3. The whole gel images for western blot results in Figure S1-4. (**a**). The

whole gel image for Fig. S1f. (**b**). The whole gel image for Fig. S1h. (**c**). The whole gel image for Fig. S2a. (**d**). The whole gel image for Fig. S2d. (**e**). The whole gel image for Fig. S2f. (**f**). The whole gel image for Fig. S2g. (**g**). The whole gel image for Fig. S2h. (**h**). The whole gel image for Fig. S2i. (**i**). The whole gel image for Fig. S3f.
(**j**). The whole gel image for Fig. S4a. (**k**). The whole gel image for Fig. S4b. (**l-n**). The whole gel image for Fig. S4c. (**o**). The whole gel image for Fig. S4d. Red dotted lines indicated the part showed in Figures.

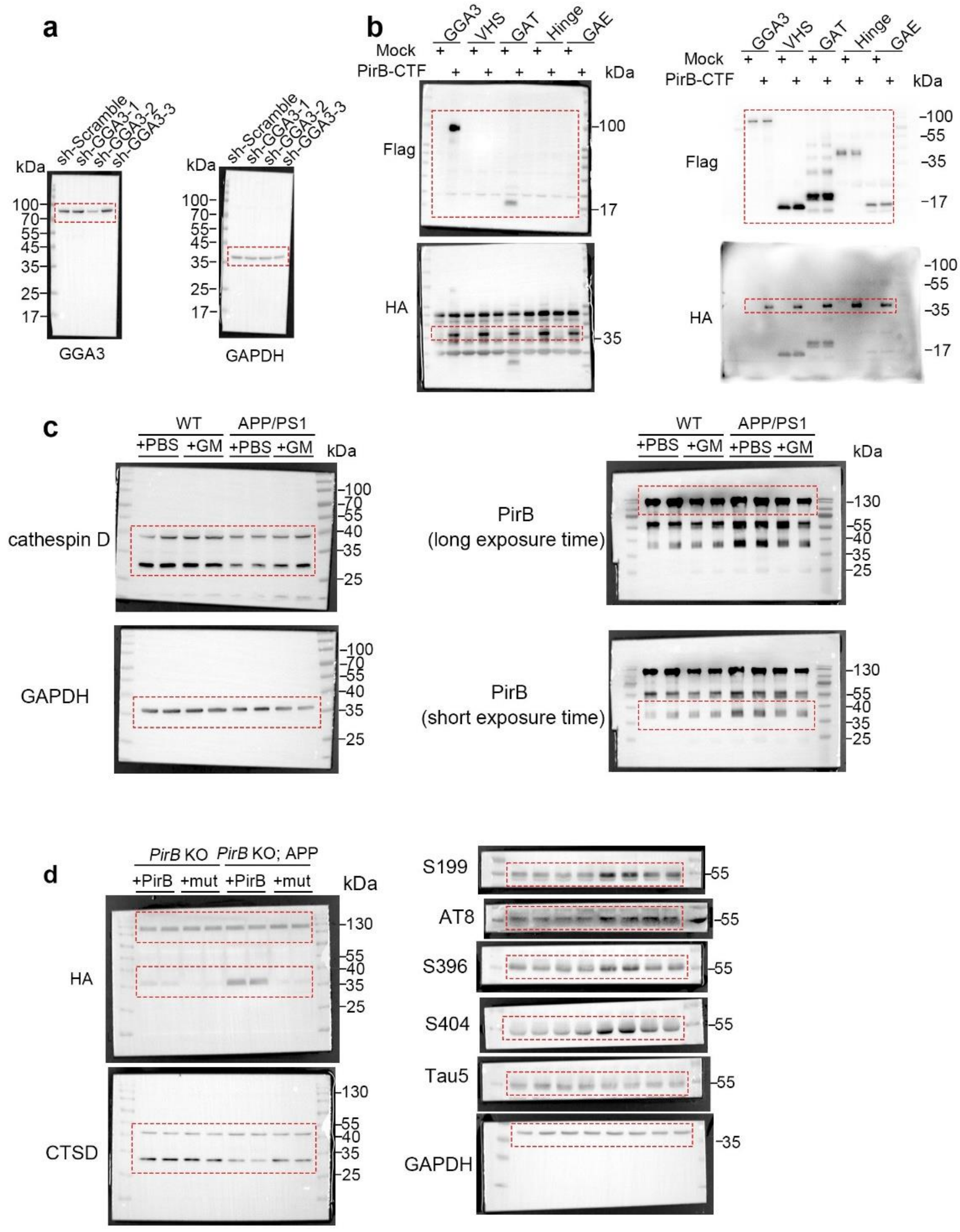

Uncut gels 4. The whole gel images for western blot results in Figure S5-6. (**a**). The whole gel image for Fig. S5e. (**b**). The whole gel image for Fig. S5g. (**c**). The whole gel image for Fig. S6d. (**d**). The whole gel image for Fig. S6f. Red dotted lines indicated

the part showed in Figures.
